# The Prediction of Biological Features Using Magnetic Resonance Imaging in Head and Neck Squamous Cell Carcinoma: A Systematic Review and Meta-Analysis

**DOI:** 10.3390/cancers15205077

**Published:** 2023-10-20

**Authors:** Hedda J. van der Hulst, Robin W. Jansen, Conchita Vens, Paula Bos, Winnie Schats, Marcus C. de Jong, Roland M. Martens, Zuhir Bodalal, Regina G. H. Beets-Tan, Michiel W. M. van den Brekel, Pim de Graaf, Jonas A. Castelijns

**Affiliations:** 1 Department of Radiology, The Netherlands Cancer Institute, 1066 CX Amsterdam, The Netherlands; 2 GROW School for Oncology and Developmental Biology, University of Maastricht, 6211 LK Maastricht, The Netherlands; 3 Department of Otolaryngology and Head & Neck Surgery, Amsterdam UMC Location Vrije Universiteit Amsterdam, 1081 HZ Amsterdam, The Netherlands; 4 Department of Radiology and Nuclear Medicine, Amsterdam UMC Location Vrije Universiteit Amsterdam, 1081 HZ Amsterdam, The Netherlands; 5 Cancer Center Amsterdam, Imaging and Biomarkers, 1081 HV Amsterdam, The Netherlands; 6 School of Cancer Science, University of Glasgow, Glasgow G61 1QH, UK; 7 Department of Radiation Oncology, The Netherlands Cancer Institute, 1066 CX Amsterdam, The Netherlands; 8 Scientific Information Service, The Netherlands Cancer Institute, 1066 CX Amsterdam, The Netherlands; 9 Department of Regional Health Research, University of Southern Denmark, 5230 Odense, Denmark; 10 Department of Head and Neck Oncology and Surgery, The Netherlands Cancer Institute, 1066 CX Amsterdam, The Netherlands; 11 Department of Otolaryngology and Head & Neck Surgery, Amsterdam UMC Location University of Amsterdam, 1081 HZ Amsterdam, The Netherlands

**Keywords:** HNSCC, radiogenomics, MRI, HPV status, Ki-67 proliferation marker, HIF-1α, DWI, DCE

## Abstract

**Simple Summary:**

This systematic review evaluates the potential of magnetic resonance imaging (MRI) to predict tumor biology in primary squamous cell carcinoma of the head and neck (HNSCC). Fifty-eight articles were analyzed, examining the relationship between MRI parameters and biological features. Most studies focused on HPV status associations, revealing that HPV-positive tumors consistently exhibited lower diffusion-weighted metrics. Moreover, lower diffusion values were also with a high Ki-67 proliferation index, indicating high cellularity. Several perfusion parameters describing the vascularity were significantly associated with HIF-1α. Analysis results of other biological factors (VEGF, EGFR, tumor cell count, p53, and MVD) were inconclusive. Larger datasets are needed to develop and validate radiomic-based prediction models, which already show promising results in capturing diverse tumor biology features. Overall, MRI holds potential for non-invasive and rapid tumor biology characterization, enhancing future clinical outcome predictions and personalized patient management for HNSCC.

**Abstract:**

Magnetic resonance imaging (MRI) is an indispensable, routine technique that provides morphological and functional imaging sequences. MRI can potentially capture tumor biology and allow for longitudinal evaluation of head and neck squamous cell carcinoma (HNSCC). This systematic review and meta-analysis evaluates the ability of MRI to predict tumor biology in primary HNSCC. Studies were screened, selected, and assessed for quality using appropriate tools according to the PRISMA criteria. Fifty-eight articles were analyzed, examining the relationship between (functional) MRI parameters and biological features and genetics. Most studies focused on HPV status associations, revealing that HPV-positive tumors consistently exhibited lower *ADC_mean_* (SMD: 0.82; *p* < 0.001) and *ADC_minimum_* (SMD: 0.56; *p* < 0.001) values. On average, lower *ADC_mean_* values are associated with high Ki-67 levels, linking this diffusion restriction to high cellularity. Several perfusion parameters of the vascular compartment were significantly associated with HIF-1α. Analysis of other biological factors (VEGF, EGFR, tumor cell count, p53, and MVD) yielded inconclusive results. Larger datasets with homogenous acquisition are required to develop and test radiomic-based prediction models capable of capturing different aspects of the underlying tumor biology. Overall, our study shows that rapid and non-invasive characterization of tumor biology via MRI is feasible and could enhance clinical outcome predictions and personalized patient management for HNSCC.

## 1. Introduction

Over the years, magnetic resonance imaging (MRI) has steadily improved in rapid acquisition, image quality, and functional imaging capabilities, rendering it indispensable for the diagnostics and follow-up of the anatomically complex and biologically heterogenous head and neck squamous cell carcinomas (HNSCCs) [1,2]. As a non-invasive and routinely used imaging technique with additional functional sequences, MRI presents a valuable modality to capture this biological heterogeneity and enable longitudinal evaluation of HNSCC [1].

In HNSCC research, biological tumor characteristics have been a frequent topic, with a notable emphasis on subtype entities based on human papillomavirus (HPV) status and tumor components like vascularization and proliferation. 

HPV-positive (HPV^+^) tumors are predominantly found in the oropharynx and are caused by the HPV-16 subtype. They are characterized by the presence of the viral proteinases E6 and E7, which ultimately leads to the degradation of p53 (tumor suppressor protein 53) and retinoblastoma 1 protein (Rb) [3,4]. In contrast, nearly all HPV-negative (HPV^−^) tumors deactivate the p53 tumor suppressor pathway via *TP53* gene mutations [4]. HPV-negative tumors are associated with worse recurrence rates, metastasis, and overall worse clinical outcomes than HPV-positive tumors [3,4]. 

Providing vascular support for tumor growth and angiogenesis is another critical factor that also influences the efficacy of radiotherapy and chemotherapy [5]. Vascular endothelial growth factor (VEGF), microvessel density (MVD), and hypoxia-inducible factor-1 alpha (HIF-1α) are histopathological markers that are used to estimate the extent of angiogenesis and to deduct the presence of hypoxia [6,7]. Furthermore, tumor cell proliferation, measured using the Ki-67 proliferation index and frequently driven by the epidermal growth factor receptor (EGFR), links those markers to tumor growth [8]. Higher tumor growth, proliferation, and angiogenesis rates are associated with increased metastatic potential, and larger tumors show worse outcomes [5,9]. On a genetic level, signatures such as epithelial-to-mesenchymal transition (EMT), tumor lymphocyte infiltration, and DNA repair deficits, are also known to affect outcomes [10]. To date, complex immunohistochemical (IHC) assays, gene expression analysis, DNA sequencing, and/or transcriptional profiling are required to identify such biological characteristics for outcome prediction or treatment optimization. 

MRI offers the means to monitor various biological processes within tumors. Notably, functional techniques like diffusion-weighted imaging (DWI) and dynamic contrast-enhanced (DCE) imaging excel in unveiling tissue microstructures, perfusion patterns, and vascular attributes [1,11]. However, morphological sequences like T1-weighted (T1W) or T2-weighted (T2W) imaging remain indispensable as these functional techniques may distort or suppress the extraction of the exact anatomical location [1]. 

Multi-sequence data can be combined for quantitative image analysis in the emerging field of radiomics. Radiomics may be deployed to predict important outcomes by defining biological or genomic factors in studies that are often referred to as radiogenomics [12,13]. Radiogenomics has been increasingly investigated, primarily using computed tomography (CT) images [14]. In HNSCC, however, the superior soft tissue depiction of MRI in combination with functional imaging is expected to provide a better representation. 

This systematic review and meta-analysis aims to assess the current level of evidence of biological feature prediction using quantitative MRI analysis. For this purpose, it evaluates the diagnostic accuracy of both stand-alone functional (diffusion and perfusion) and anatomical (T1W, T2W) MRI parameters, along with the utility of multi-feature radiomics models.

## 2. Materials and Methods

This systematic review (PROSPERO registration: 392509) was performed following the Preferred Reporting Items for Systematic Reviews and Meta-Analysis (PRISMA) criteria [15]. 

### 2.1. Search Strategy

A comprehensive search was conducted in PubMed (MEDLINE), Embase, and Scopus for original articles published until 28 July 2023. The search query consisted of “Head and neck squamous cell carcinoma” combined with “MRI”, along with either “Genes”, “HNSCC tumor Biomarkers”, or “radiogenomics”, along with their synonyms or adjacent terms. 

The literature search is explained in Supplementary Material S1, Appendix S1, and the full search for the different databases is provided in Supplementary Material S1, Appendix S2. Two researchers (BLINDED and BLINDED) independently screened the results for relevant publications using the Rayyan web application [16]. The reviewers discussed and resolved discrepancies during multiple consensus meetings following the screening of the same subset of items.

### 2.2. Study Selection

The following selection criteria were applied by the reviewers: (1) human subjects were examined; (2) only original research was considered; (3) the research included MRI parameters of the primary tumor; (4) the study compared quantitative MRI parameters with genetics/histopathology markers; (5) the study solely included HNSCCs at any age, gender, and stage; (6) the study was not a case report; (7) and it was written in the English language. 

### 2.3. Quality Assessment

An optimized version of the Quality Assessment of Diagnostic Accuracy Studies 2 (QUADAS-2) tool [17] was used to assess the “applicability concerns” and “risk of bias” in the remaining eligible articles (see Supplementary Material S1, Appendix S3). The various biological features were assessed separately to take into account the varying measurement characteristics, often necessitating multiple independent assessments of the same article. 

Articles reporting radiomic models were additionally scored using the radiomics quality score (RQS) to evaluate the quality, design, and generalizability of the model [18].

### 2.4. Data Extraction and Analyses

Data were extracted using a custom-built standardized extraction form by one reviewer (BLINDED). Collected data included study characteristics (e.g., study design), patient characteristics (e.g., gender and cancer subsite), methods used for biological feature evaluation (e.g., DNA polymerase chain reaction (PCR), immunohistochemistry (IHC), and the number of observers for IHC methods), techniques and its properties used for imaging evaluation (e.g., MRI field strength, use of diffusion-weighted and/or perfusion-weighted imaging, and delineation procedure and characteristics), and statistical methods adopted to compare the MRI features with the biological endpoints. 

The results from biological factors analyzed in a reasonable number of studies, determined as four or more studies, are compared and summarized in the main text of this review. Survey results of those with fewer studies are listed in the Supplementary Materials. 

When comparable data were available for a considerable number of studies, a set of four or more studies, a meta-analysis was conducted. For this reason, either the standardized mean difference (SMD, Cohen’s d [19]) or the correlation [20] with their 95% confidence intervals (95%CI) were calculated for each study and visualized in a forest plot. If a mean value of the imaging parameter was not provided but a median value was reported, we estimated the mean value using the formulas from Lou et al. and Wan et al. after confirming that the data were not too skewed [21,22,23,24]. Supplementary Material S1, Appendix S4, outlines all the statistical formulas used in this study. If data were unsuitable for meta-analyses, a narrative summarization of the available data was provided. In all analysis, *p*-values < 0.05 were considered as statically significant.

## 3. Results

### 3.1. Literature Search 

**Figure 1:**
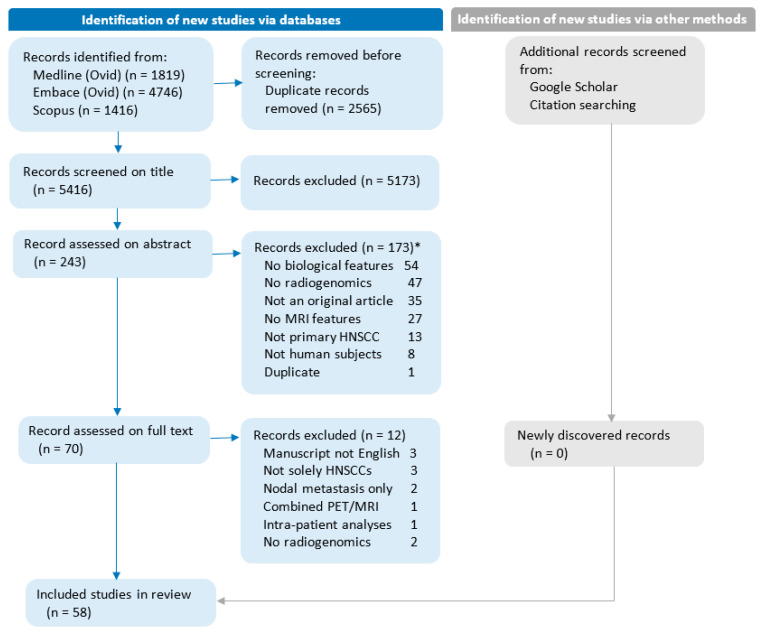
Flow diagram of the study selection and exclusion process. No radiogenomics: Studies without analyses of biological features compared with MRI features. Combined PET/MRI: Studies focusing on combined PET/MRI parameters rather than stand-alone MRI parameters. Intra-patient analyses: Studies are limited to analyzing multiple biopsies within the same patient. * Studies can be excluded for more than one reason.

A literature search was conducted to find studies reporting biological and MRI feature associations and produced a total of 5396 unique entries. Most studies were excluded based on title (*n* = 5173, 95.5%) or abstract (*n* = 173, 3.2%) evaluation. Full-text evaluation was performed for 70 entries (1.2%), of which 12 were excluded (17% of 70). Figure 1 provides a flow diagram of the study selection and exclusion process, detailing the reasons for exclusion. Finally, a total of 58 studies [25,26,27,28,29,30,31,32,33,34,35,36,37,38,39,40,41,42,43,44,45,46,47,48,49,50,51,52,53,54,55,56,57,58,59,60,61,62,63,64,65,66,67,68,69,70,71,72,73,74,75,76,77,78,79,80,81,82] satisfied all inclusion criteria. 

Two articles compared genetics and radiomic features, aligning with the research objectives [76,77]. Regarding individual biological endpoints, HPV status, Ki-67, tumor cell count, HIF-1α, VEGF, EGFR, p53, and MVD were each covered by at least four original articles, which together constituted a total of 51 articles. These data provided sufficient material for data analysis, as defined in Section 2, and are evaluated in detail below. The five remaining articles focused on the Epstein–Barr virus (EBV) [78], proliferating cell nuclear antigen (PCNA) [79], carbonic anhydrase 9 (CAIX) [80], tumor–stroma ratio [81,82], or tumor-infiltrating lymphocytes [82]. Supplementary Material S2, Appendix S4, provides an overview of all identified biological endpoints along with the amount of significant correlations or differences reported in these studies.

### 3.2. Quality Assessment

Quality assessment and applicability analyses were performed using the QUADAS-2 tool [17] and are shown in Supplementary Material S2, Appendices S1 and S2. Across all studies, “reference standard” applicability concerns were most frequently recorded, while “patient selection” and “index test” were acceptable in most studies. Applicability concerns derived from mismatched research questions (i.e., studies including nodal metastasis, radiogenomics not being the main research question, or use of cut-off values for biological factor analyses). Concerns for bias were related to “flow and timing” issues, with discrepancies in MRI-to-biopsy timelines or varied MRI protocols applied. In the “reference standard” category, potential bias could arise from single IHC reviewer use or whether a continuous or cut-off value was used when reporting a biological factor. Table 1 and Table 4 display the results from the RQS evaluation [18] for all radiomic model studies, revealing deficiencies in open science practices, validation approaches, and clinical utility.

### 3.3. Study Outcome Assessment 

The reported MRI and biological feature association study results were structured into HNSCC tumor biology categories. Meta-analyses were performed where applicable. The majority of the MRI-based radiomic studies in HNSCC were focused on links with HPV status, allowing for more extensive cross-study comparisons. Analysis of associations with proliferation, vasculature, and perfusion-related markers was the focus of the assessment of the remainder of the studies. 

#### 3.3.1. Human Papilloma Virus (HPV)

HPV status plays a pivotal role in HNSCC staging and outcome prediction. Twenty-nine studies [25,26,27,28,29,30,34,35,36,37,38,43,44,45,46,47,48,49,52,53,54,55,56,57,58,59,69,70,74] involving 2122 patients tested whether HPV status can be deducted using quantitative imaging analysis. 

##### HPV: Independent T1W and T2W Texture Parameters and Radiomic Models

As a standalone parameter, tumor volume was analyzed for associations with HPV status in five studies [35,38,45,56,69] (Table 1). None of these studies observed significant differences in tumor volumes between HPV^+^ and HPV^−^ tumors (Supplementary Material S2, Appendix S3 (A3.1)).

Only one (Giannitto et al. [37]) out of four studies [37,43,49,55] reported a significant association between T1W and T2W MRI stand-alone histogram features and HPV status. Analyzing 1286 radiomics features, Giannitto et al. [37] suggested that HPV^−^ tumors had a more varied texture than HPV^+^ oropharyngeal HNSCC, providing the basis for the observed association with stand-alone histogram parameters [37]. 

MRI radiomic-based prediction models for HPV status were built in eight studies [26,27,28,46,53,58,59,74] (Table 1). These studies based their analysis exclusively on oropharyngeal SCC of different stages. Marzi et al. [46] constructed a model with three DWI or intravoxel incoherent motion (IVIM) features out of the 157 extracted features to predict HPV. However, its diagnostic performance decreased after internal validation (AUC 0.91 to 0.66) [46]. A model starting with 498 native 2D T1W features [26] and trained on images from 249 patients achieved a 5- and 10-fold cross-validation AUC of 0.79 in its training set, yet no validation was conducted. Three studies [27,28,53] employed 3D T1W imaging with gadolinium-based contrast, yielding in-hospital validation AUCs of 0.76 to 0.83 in their respective test sets. In both Sohn et al. and Li et al.’s studies, feature selection of the six most relevant contrast-enhanced T1W and T2W features yielded models with an AUC of 0.74 in their respective test sets [58,74]. Additionally, another multi-sequence model, incorporating potentially native T1W, contrast-enhanced T1W, T2W, and apparent diffusion coefficient (ADC) features, resulted in similar AUC values of 0.76 [59]. Among the combined features, six ADC histogram features and one native T1 feature were identified as the best combination within these analyzed data [59]. None of the models were tested in independent validation or external data sets. 

Forest plots of standard mean differences (SMDs) in mean ADC values of HPV-positive and HPV-negative tumors were grouped according to HPV determination methodology. Conventional HPV diagnostics using p16 IHC combined with PCR were used in seven studies. One single assay was used in the remainder (p16 IHC, HC2 High-risk HPV DNA test, or hybrid capture assay kits). In studies that provided median values, standard mean differences were calculated using estimated means as indicated (and as described in Section 2 and Supplementary Material S1, Appendix S4). 

##### HPV: Diffusion-Weighted Imaging

DWI features in relation to HPV status were analyzed in sixteen studies [29,34,35,36,38,43,44,45,46,47,52,54,55,57,69,70], encompassing a total of 897 patients (range n: 20–105), predominantly with oropharyngeal SCC (78.4%) and with a similar average age of 63. Twelve of the articles reported on retrospectively collected data. ADC mapping was acquired using b0 and b1000 in eight of the sixteen studies. Table 2 highlights the key characteristics of the included studies. 

**Figure 2:**
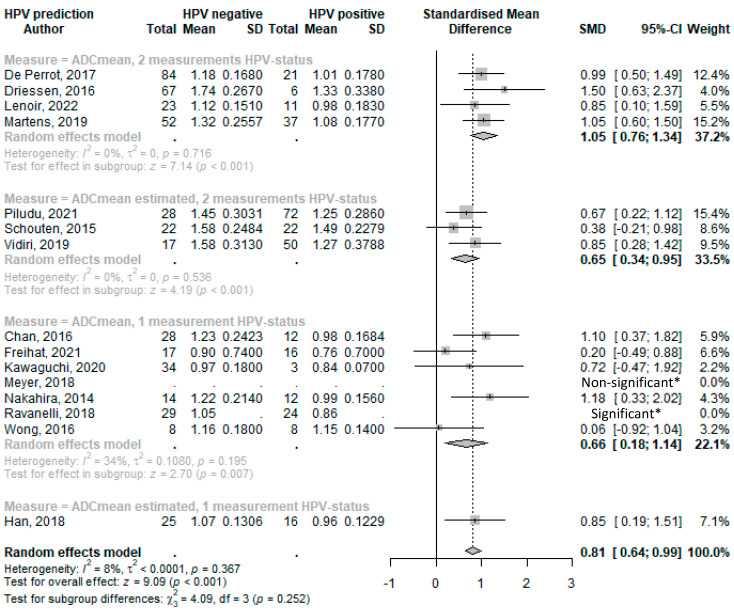
Forest plot of the mean ADC values with HPV status. * Limited data available and as reported in reference. Abbreviations: SD = standard deviation; SMD = standard mean difference; and CI = confidence interval [34,35,44,45,54,57,69,29,36,43,47,52,55,70,38].

**Figure 3:**
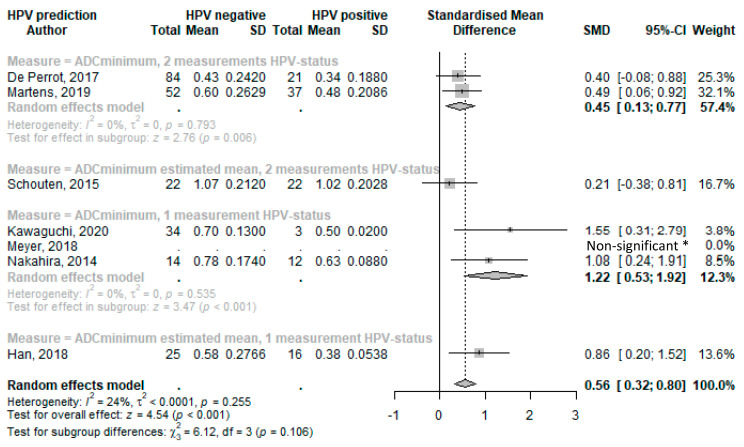
Forest plot of the minimal ADC values with HPV status. * Limited data available and as reported in reference. Abbreviations: SD = standard deviation; SMD = standard mean difference; CI = confidence interval [34,45,57,43,47,52,38].

From all analyzed DWI parameters, and with fifteen and seven studies, respectively, *ADC_mean_* and *ADC_mininum_* were the most reported. *ADC_mean_* and *ADC_mininum_* standardized mean differences between HPV^+^ and HPV^−^ as reported or as deduced from estimated mean values (Supplementary Material S1, Appendix S4) are displayed in Forest plots in Figure 2 and Figure 3. Standard mean difference (SMD) analyses are grouped according to HPV determination accuracy (Table 2) as HPV detection accuracy increases when applying combined p16-IHC and HPV16 and HPV18 DNA or E6/7 RNA PCR tests [83]. 

The Forest plot analyses (Figure 2) revealed that HPV^+^ HNSCC has a lower average *ADC_mean_* value compared with HPV^−^ subtypes, with an overall SMD of 0.81 (95%CI 0.64–0.99; *p* < 0.001). Notably, and consistent with an increased specificity, this difference was more pronounced when HPV status was ascertained by using two different HPV determination methods (SMD: 1.05, 95%CI 0.76–1.34; *p* < 0.001). However, the difference and association were weaker when relying on a single HPV detection method (SMD: 0.66, 95%CI 0.18–1.14; *p* = 0.007) or on estimated means that generally show smaller SMDs.

Forest plots of standard mean differences in minimal ADC values of HPV-positive and HPV-negative tumors were produced and grouped according to HPV determination methodology. Conventional HPV diagnostics using p16 IHC combined with PCR were used in three studies. One single assay was used in the remainder (either p16 IHC or hybrid capture assay kits). In studies that provided median values, standard mean differences were calculated using estimated means as indicated (and as described in Section 2 and Supplementary Material S1, Appendix S4). 

The overall random effect model of all studies that used *ADC_minimum_* to predict HPV status resulted in a statistically significant SMD of 0.56 (95%CI 0.32–0.80; *p* < 0.001) (Figure 3). The *ADC_minimum_* SMD values for HPV determined with two different HPV determination methods showed a lower effect compared with the overall values, with an SMD of 0.45 (95%CI 0.13–0.77; *p* = 0.006) for studies that provided means, and an SMD of 0.21 (95%CI −0.38–0.81) for the study that provided medians (of which we estimated means). *ADC_minimum_* with only one measurement of HPV showed a higher SMD of 1.22 (95%CI 0.53–1.92; *p* < 0.001) for the study that provided a mean, and an SMD of 0.86 (95%CI 0.20–1.52) for studies with an estimated mean. 

Three studies [46,54,69] that also evaluated IVIM parameters found significantly lower true diffusion coefficient (D_t_) values in HPV^+^ patients compared with HPV^−^ patients when using higher b-values (b300–b800). This association exhibited overall lower *p*-values than their reported *ADC_mean_* analyses (*p* <0.001–0.001). 

Among the other frequently reported ADC metrics, the histogram-based features of skewness and kurtosis were analyzed in five studies [34,44,45,47,55]. Only two studies [34,44] reported significant differences and higher skewness and kurtosis values for HPV^+^ tumors. These and other associations can be found in Supplementary Material S2, Appendix S3 (A3.2).

##### HPV: Perfusion-Based Imaging

The mean values of the DCE parameters, *K^trans^*, *K_ep_*, and *V_e_* were reported by four studies [30,38,48,54]. However, the findings comparing HPV status between groups were inconsistent across these studies. Significant differences in these parameter values were reported in only one study by Choi et al. [30], who reported a significantly higher mean *K^trans^* for HPV^+^ tumors compared with HPV^−^. This finding, however, contrasts with the other studies [38,54] that show (non-significantly) lower *K^trans^* values in HPV^+^ tumors. 

In addition to mean DCE parameter values, Choi et al. and Meyer et al. also analyzed histogram parameters of *K^trans^*, *K_ep_*, and *V_e_* as 25th, 50th, and 75th percentiles (P), skewness, and kurtosis [30,48]. Significantly higher values for the P25, P50, and P75 of the *K^trans^* and the P25 of the *K_ep_* were found for p16 IHC-positive compared with p16 IHC-negative tumors in Choi et al. These findings were not replicated by Meyer et al. Additional reported associations can be found in Supplementary Material S2, Appendix S3 (A3.3).

#### 3.3.2. Tumor Cell Proliferation and Cellularity Markers: Ki-67, EGFR, Tumor Cell Count, and p53

MRI parameter associations with tumor cell proliferation or density-related markers were discussed in 19 studies, including Ki-67, EGFR, tumor cell count, and p53.

##### Ki-67 Proliferation Index 

The Ki-67 proliferation marker is a reliable IHC method for determining tumor cell proliferation. Associations between the Ki-67 proliferation marker and MRI parameters were assessed in fifteen studies (Table 3). 

**Figure 4:**
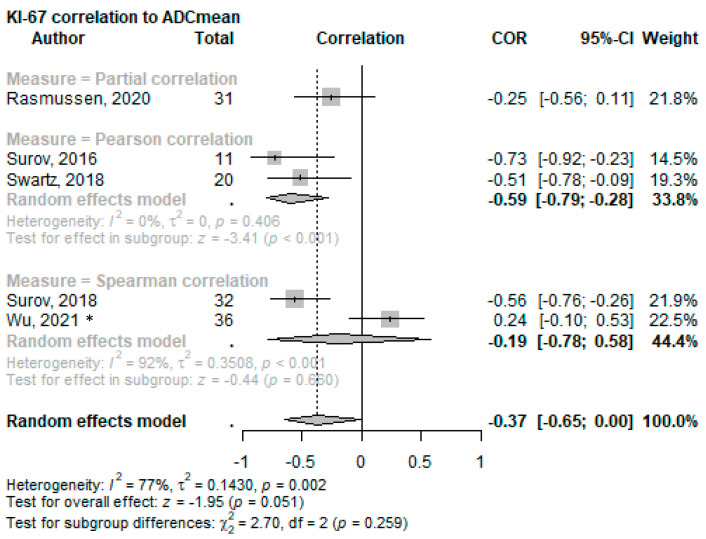
Forest plot of the correlation of mean ADC values with the Ki-67 proliferation index split using the correlation test applied. * This cohort consists solely of nasopharyngeal squamous cell carcinoma [60,61,65,63,71].

The associations between the Ki-67 proliferation index and the DWI-metric *ADC_mean_* were examined in six studies [60,61,63,65,71,72]. Four studies [60,61,63,65] directly investigated the correlation and found a significant inverse association (partial correlation *r* = −0.253 to *r* = −0.728; *p* < 0.001–0.024). In contrast, Wu et al. [71] reported a positive but non-significant correlation between *ADC_mean_* and Ki-67 (*r* = 0.238; *p* = 0.163). An overall effect of COR (−0.37, 95%CI −0.65–0.00; *p* = 0.051) was calculated; see Figure 4 for the forest plot. Additionally, Shima et al. [72] reported lower *ADC_mean_* values for a higher Ki-67 index (*p* = 0.012).

*ADC_maximum_* values also significantly correlated with Ki-67 (ρ = −0.46 to ρ = −0.640; *p* = 0.0079 to 0.036) in two studies [61,63], while the correlation with *ADC_minimum_* was significant in just one [63] of the three [61,63,71] reporting articles (ρ = −0.58; *p* = 0.0005). Two studies utilized the diffusion kurtosis imaging (DKI) technique, reporting different outcome parameters, but both highlighted the potential value of DKI in evaluating Ki-67 expression [72,73]. Further details on these associations can be found in Supplementary Material S2, Appendix S3 (A3.3). 

Out of the five articles [40,41,60,62,64] that analyzed the correlation of Ki-67 with the DCE parameter mean *K^trans^*, only one study by Surov et al. [62] found significant correlation with Ki-67 (*r* = −0.62, *p* = 0.041). The four reporting articles found no significant correlation with Ki-67 for the mean *K_ep_* and mean *V_e_* [40,41,62,64]. IVIM parameter analysis also did not reveal significant associations with the Ki-67 proliferation index [71].

##### Epidermal Growth Factor Receptor (EGFR)

Cellular signaling from the epidermal growth factor receptor (EGFR) induces cell proliferation and is frequently overexpressed or mutated in HNSCC [84]. Seven articles [30,40,47,48,49,60,67] assessed associations between histopathologically determined EGFR expression and MRI parameters, but no consistent results were found (See Table 3 for study characteristics).

No significant relationship was found between the EGFR and T1W or T2W imaging [49], *ADC_mean_* [47,60,67], or any other histogram parameter of the ADC map [47].

Mean *K^trans^* results were inconsistent in the four publications [30,40,48,60] examining associations between EGFR and DCE parameters. One study [40] reported a significantly higher mean *K^trans^* value in the EGFR overexpressing tumor group (*p* < 0.0001), while another study [30] found this to be significantly lower (*p* = 0.047). The remaining two papers [48,60] found no significant association between mean *K^trans^* and EGFR overexpression status. Similar inconsistencies were seen for *K_ep_*; Choi et al. [30] reported a negative association with significantly lower *K_ep_* values for EGFR overexpression (*p* = 0.004), while Huang et al. [40] reported non-significant but higher *K_ep_* values for high EGFR expression.

##### Tumor Cell Count

Six studies [49,60,61,62,63,64] investigated potential associations between pathologically assessed tumor cell count and MRI parameters with moderate success. MRI perfusion studies did not reveal any significant correlations between mean *K^trans^* or mean *K_ep_* and *V_e_* and tumor cell counts [60,62,64]. Only one [63] of the three MR diffusion studies [60,61,63] showed a significant moderate inverse correlation between *ADC_mean_* and tumor cell count (ρ = −0.56; *p* = 0.0009). *ADC_minimum_* also exhibited a significant positive correlation with cell count in the same study (ρ = −0.60; *p* = 0.0003) [63]. See Supplementary Material S2, Appendix S3, for all non-significant tested associations.

##### Tumor Suppressor Protein p53

Tumor suppressor protein p53 regulates cell cycle progression, and its expression is either affected by HPV in HPV^+^ HNSCC or by TP53 mutations exclusively found in HPV^−^ HNSCC [3,4]. 

Five studies [32,47,48,49,60] attempted to link MRI parameters with p53 status in HNSCC. Dang et al. [32] used DWI and contrast-enhanced T1W and T2W features of the oropharyngeal SCC (OPSCC) and classified p53-positive (p53^+^) and p53-negative (p53^−^) HNSCC with 81.3% accuracy. Rasmussen et al. analyzed perfusion (mean *K^trans^*) and diffusion (*ADC_mean_*) parameters in a mixed HNSCC group that included 14.3% OPSCC and 24% lymph nodes and observed positive correlations between the p53 percentage values in both *K^trans^* (partial correlation: 0.193; *p* = 0.015) and *ADC_mean_* (partial correlation *r* = 0.190; *p* = 0.010) [60]. However, these findings for *K^trans^* and *ADC_mean_* were not replicated by Meyer et al. [47,82]. Considering HPV status, as determined using p16 IHC, Meyer et al. published three articles [47,49,82] on p53 status prediction, each using a different MRI modality: T1W or T2W [49], DWI [47], and DCE [48]. In the p53^−^ and thus the HPV^−^-enriched subgroup of Meyer et al., significant negative correlations were observed between lower *ADC_maximum_*, *ADC*-P75, *ADC*-P90, *ADC*-SD [47], *V_e_*max [82] and a larger positively stained p53 expression (ρ = −0.827; *p* = 0.002, ρ = −0.763; *p* = 0.01, ρ = −0.836; *p* = 0.001, ρ = −0.70; *p* = 0.016, ρ = −0.80; and *p* = 0.009, respectively). Additionally, T2Wmax (ρ = 0.736; *p* = 0.015), T2W-P90 (ρ = 0.68; *p* = 0.028), and T2W-SD (ρ = 0.760; *p* = 0.011) [49] positively correlated with the stained p53 expression. Within the p16 positive and thus likely HPV-positive oropharyngeal subgroup, higher T2W-mean, T2W-P25, T2W-P75, T2W-median, and T2W-mode [49] were positively associated with higher p53 staining (ρ = 0.569; *p* = 0.007, ρ = 0.508; *p* = 0.019, ρ = 0.479; *p* = 0.028, ρ = 0.555; *p* = 0.009, ρ = 0.468; *p* = 0.033, respectively). Lower *ADC*-kurtosis (ρ = −0.446; *p* = 0.029) [47] and T1W-entropy (ρ = −0.648; *p* = 0.001) [49] demonstrated a negative association with highly stained p53 expression. 

None of the included studies counted the complete absence of p53 expression as a mutation. 

#### 3.3.3. Tumor Vasculature: HIF-1α, VEGF, and MVD

Biological features that impact or reflect tumor vasculature, such as the microvessel density (MVD), hypoxia markers such as HIF-1α, or angiogenesis-promoting vascular endothelial growth factor (VEGF), are bound to affect diffusion and perfusion parameters as determined using functional MRI and have, therefore, been the subject of “radiogenomic” studies [5,6,7].

##### Hypoxia-Inducible Factor (HIF)-1α

The association of HIF-1α with MRI parameters was addressed in eight publications [31,39,40,42,47,48,49,65] (See Table 3 for all study characteristics). 

One study by Chen et al. determined localized higher expression of HIF-1α in tumor cells surrounding MRI-determined necrotic areas. A radiologist identified these areas using gadolinium contrast-enhanced T1W and T2W MRI [31]. Meyer et al. [49] observed a significant negative correlation between several standalone T2W imaging histogram parameters and HIF-1α in p16-negative HNSCC (Supplementary Material S2, Appendix S3 (3A.1)). 

Three studies [39,40,42] analyzed the volume and speed of the blood running through the tissue using different metrics: fractional plasma volume (*V_p_*) (*r* = 0.173; *p* = 0.327) [39]; tumor blood flow (*TBF*) (*p* < 0.001); fluid flow velocity (|ⴎ|) (*p* < 0.001) [40]; and maximum enhancement (*ME*) (*p* = 0.001), relative enhancement (*RE*) (*p* = 0.027), and maximum relative enhancement (*MRE*) (*p* = 0.017) of the time–signal intensity curve [42]. Most of these parameters showed either a positive correlation or a significantly higher value for higher HIF-1α expression, except for *V_p_* as measured by Hu et al. Yet other DCE parameters describing the extravascular extracellular space (EES) (e.g., *V_e_*) or the transfer between the two compartments (*K^trans^*, *K_ep_*) did not have significant correlations [39,40,48].

##### Vascular Endothelial Growth Factor (VEGF)

VEGF was analyzed in eight original articles [33,39,47,48,49,60,66,67]. 

Positive correlations were reported for the T1W-P10 histogram parameter [49] (ρ = 0.371, *p* = 0.04) and vascular compartment parameter *V_p_* (*r* = 0.339; *p* = 0.05) in [39]. A similar blood volume (*V_b_*) parameter measured through a two-compartment model applied by Donaldson et al. [33] did not replicate this. Donaldson et al. found a significant strong inverse relationship between *F_b_* (whole-blood perfusion) and VEGF expression using PCR-derived VEGF mRNA measures (*r* = −0.82; *p* = 0.023) [33]. For more details on the non-significant associations tested, refer to Supplementary Material S2, Appendix S3.

##### Microvessel Density (MVD)

The variability of molecular markers used for microvessel identification and assessment, such as CD105 [41,48,51], CD34 [39,41,68], and CD31 [62,66], hampered comparisons across the seven studies that investigated correlations between MVD and MRI parameters (Table 3). 

DCE parameters (mean *K^trans^*, *K_ep_*, and *V_e_*) were analyzed in four studies [39,41,50,62]. One paper by Meyer et al. [50] revealed a significant positive correlation between high mean *K_ep_* and a higher number of CD105-derived microvessels. At the same time, another article by Karabay et al. [41] indicated a positive correlation between mean *K^trans^* and the number of CD34-derived microvessels (*r* = 0.346; *p* = 0.049) but not for the CD105-derived count (*r* = 0.307; *p* = 0.08). No other discernable trends or correlations were observed in the other studies [39,62]. Unetsubu et al. and Tekiki et al. examined correlations between CD34 and CD31 MVD, respectively, and contrast index (CI) parameters. Both studies observed a significant positive correlation between MVD and CI-gain, representing the maximum gradient during the upslope phase of the enhancement curve (*r* = 0.46 to *r* = 0.49; *p* = 0.00821 to *p* = 0.037). No significant correlation was found between MVD and CI-max, representing the maximum amplitude of contrast enhancement [66,68].

When investigating the stained vessel area as calculated using either CD105 [50] or CD31 [62], only Surov et al. uncovered a significant positive correlation with mean K_ep_ (*r* = 0.67; *p* = 0.02) [62], while Meyer et al. did find other correlations with K_ep_ histogram parameters [50] (see Supplementary Material S2, Appendix S3 (A3.3)). 

#### 3.3.4. Radiomics and Genomics Linkage Studies

Two articles [76,77] included broader genomic analyses. Both articles focused on developing radiomic-based treatment outcome prediction models using datasets as outlined in Table 4. Separate, much smaller cohorts, consisting of 9 [76] to 16 [77] patients with gene expression data, were used to explore potential relationships with such biological endpoints. Please note that these separate analyses strongly lack statistical power. 

**Table 4:** Radiomic prediction model study design characteristics with their radiomics quality score.

Radiomic Models for Biological Signature											
Study, Year	Location Inclusion Center	Train (N)	Test (N)	Age (mean)	Male (%)	Tumor Subside	Tumor Stage	Modality	#Features	Total RQS	Domains: IM/ FR/VA/PI/LE/OS
Gao, 2021 [76]	Hunan, CHN	237	79	47.9	69.9	NA	All	T1+c	530	16	8/5/6/3/6/0
Zhang, 2020 [77]	Zuhai, CHN	220	44 + 44 *	47.4 †	72.7	NA	All	T1(c), T2	2364	19	8/6/6/5/7/0

The table lists study design characteristics, patient numbers, and total and individual domain quality scores of radiomics-based prediction model studies that were designed to align with biological features. Abbreviations: No. = number of selected patients; Train = number of patients analyzed in the training cohort; Test = number of patients analyzed in the test cohort; #Features = number of features collected; NA = nasopharynx; RQS = radiomics quality score; RSQ domains = IM: image protocol and feature reproducibility, FR: feature reduction and validation, VA: biologic/clinical validation and utility, PI: performance index, LE: high level of evidence, OS: open science. Note: † Median value; * 44 patients of an internal validation cohort and 44 in a separate external validation cohort.

Gao et al. [76] used their newly developed radiomic signature based on T1W with gadolinium features to predict progression-free survival (PFS) risk in a genetic data-equipped subcohort of nine patients. The genes of patients with lower PFS risk were compared with those with higher PFS risk. Furthermore, all genes were correlated to the RAD score. In a similar approach, Zhang et al. [77] initially developed a radiomic model using T1W with and without gadolinium-based agents and T2W imaging to predict failure-free survival (FFS) that was then reduced to twelve key radiomic features. This model was tested against gene mutation data in 508 genes in 16 patients. While the authors report that some texture features were associated with the chromatin remodeling pathway and higher mutational burden, the study size was too small to appropriately adjust for multiple testing, false positive rates, or to conduct multivariate analysis with other (clinical) factors. In contrast to MRI, patient outcome is defined by multiple biologic factors which likely affect the association studies in both reports. 

## 4. Discussion

**Figure 5:**
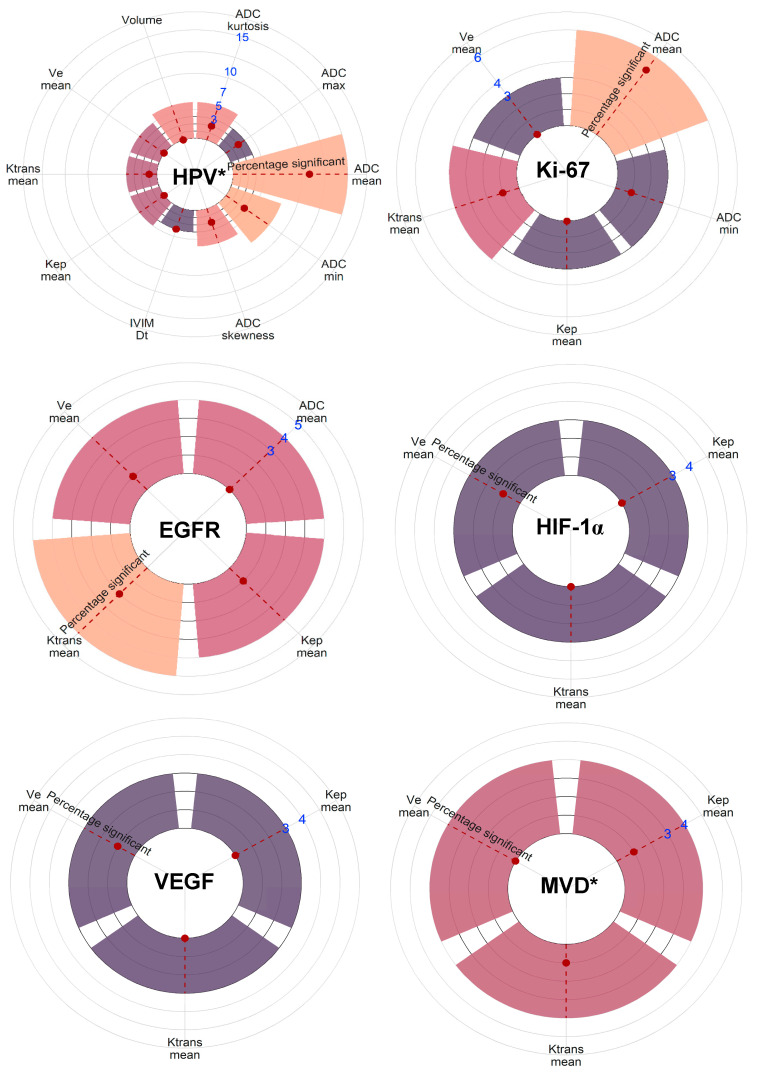
Visual representation of the number of reporting studies and the level of significance of all MRI parameters sorted by biological factor. MRI parameters are included when three or more studies have reported on associations with the specific metric and the biological factor. The count of studies is indicated by the blue numbers. The statistical significance as reported in the listed studies is indicated by the red dot on the red-striped line within the bars. * As grouped for all biological measurements defining the factor. Note: p53 lacked enough comparable studies.

MRI, with its superior soft tissue definition and functional imaging capabilities, has become a routine and indispensable tool in staging HNSCC [1]. This systematic review evaluated the current investigation status and evidence for the applicability of quantitative MRI techniques to assess the biological characteristics of primary HNSCC. Additionally, it delved into current research examining radiomic models in this context. Key findings include significantly lower *ADC_mean_* (SMD: 0.82; *p* < 0.001) and *ADC_minimum_* (SMD: 0.56; *p* < 0.001) values for HPV^+^ HNSCC compared with HPV^−^ tumors as described in multiple studies. Moreover, we repeatedly reported correlations between low *ADC_mean_* values and high levels of the proliferation marker Ki-67 (COR: −0.37; *p* = 0.051). Furthermore, functional MRI perfusion parameters that depict increased blood plasma volume and flow showed significant associations with higher HIF-1α. Figure 5 provides a visual overview of the level of significance of all MRI parameters sorted by biological factor.

### 4.1. Human Papilloma Virus (HPV)

The HPV status of the tumor emerged as the most frequently investigated endpoint, given its significant impact on prognosis and treatment decisions. Consistent with Payabvash et al.’s meta-analysis [85], the comprehensive analyses of the data in this review confirm a link between DWI parameters and HPV status. DWI-derived mean and minimum ADC values seem to be significantly lower in HPV^+^ tumors compared with HPV^−^ tumors. DWI is a functional MR technique that can assess the random movement of water molecules in the tissue microstructure, with the ADC being a derived metric used to evaluate it [47,65,86]. Hypercellular tissue is in general characterized by a low ADC value due to the restrictions on water movement imposed by the cell walls. This results locally in low water diffusivity. Conversely, local hypo-cellular tissue (e.g., tumor areas with necrosis) has a (slightly) higher ADC value due to fewer cells per volume, allowing more water movement [86,87]. 

Histopathologically, HPV^+^ OPSCCs are characterized by immature, ellipsoidal nuclei with more frequent mitosis, a high nuclear-to-cytoplasmic ratio, and decreased keratinization around the tumor periphery, possibly explaining the lower ADC values due to increased cellularity with decreased water diffusivity. HPV^−^ OPSCC typically displays better-keratinized cells with distinct cell borders and a larger amount of cytoplasm [2,3,88,89]. 

In contrast to this observation, five studies [36,43,47,57,70] reported a higher mean ADC value for HPV^+^ tumors but this did not reach significance. Possible reasons include limiting the cohort to only the biologically dissimilar nasopharyngeal SCCs (NSCCs) [43], a relatively small sample size [70], a different DWI-acquisition technique (PROPELLER), and higher b-values for the ADC calculation applied [57]. Positive p16 IHC combined with PCR detection of HPV-DNA or RNA increases specificity compared with stand-alone detection methods like p16 staining [90,91]. Incorrectly classified HPV-negative patients in studies that solely use p16 IHC in an already mixed cohort [47] may have also strongly contributed to this discrepancy. Notably, we found that reported significance values may have been erroneous in one study [36], likely based on the misreporting of some values (standard deviation did not match the reported range or *p*-value); Freihat et al.’s Student’s t-test results, which determined significant difference in *ADC_mean_* between HPV^+^ and HPV^−^ tumors, could not be replicated by us. 

In the current set of available studies, MRI perfusion parameters did not correlate with HPV status in a reproducible and consistent manner [30,38,48,54], implying that differences in the DCE parameters for HPV status are less likely to exist, thus suggesting similar vascular biology. More extensive, radiomic methods to distinguish HPV status appear promising, especially when combined with clinical parameters as they may better reflect the diverse biological differences between tumor classes. Importantly, however, as for now, none of the researched radiomic models have been externally validated. Some studies either showed a significant drop-off in AUC after an internal validation on test sets [46] or solely relied on cross-validation values instead of internal validation using independent test sets [26], raising concerns about the robustness of the models. Large, multicenter databases will be needed to build and test definitive models. 

In summary, when comparing HPV^+^ and HPV^−^ OPSCCs, HPV^+^ tumors show similar perfusion MRI parameters to HPV^−^ but dissimilar diffusion MRI parameters with consistently lower ADC values in DWI. 

### 4.2. Tumor Cell Proliferation and Cellularity Markers: Ki-67, Tumor Cell Count, and EGFR 

Cancer development and progression rely significantly on cellular proliferation. Cytotoxic drugs used in cancer therapy target highly proliferative and regenerating cells. Tumor cell proliferation markers, such as Ki-67, tumor cell count, and the EGFR, therefore play critical roles in cancer research, either as potential treatment effect monitors or therapeutic targets [92,93,94,95,96,97,98]. 

The fraction of Ki-67 positive cells, as determined using IHC, is a frequently used proliferation marker [96]. High Ki-67 expression has been correlated with poor prognosis and increased risk of lymph node metastasis [92]. Being able to monitor tumor cell proliferation through medical imaging techniques can aid in choosing and, when needed, intensifying treatments for high-proliferating tumors. 

As described in this review, the DWI parameter *ADC_mean_* is inversely correlated with the proliferation index Ki-67 and, to a lesser extent, with tumor cell count, likely due to reduced extracellular space [87]. However, a contradictory study by Wu et al. [71] reported a higher *ADC_mean_* for higher Ki-67 values, albeit insignificantly. A positive correlation between *ADC_mean_* and Ki-67 is unlikely in any tumor type, suggesting that these results may represent an outlier [99]. Yet if assuming this deviation is significant, this discrepancy could potentially be attributed to the different clinical behavior, epidemiology, histopathological characteristics, and biology of nasopharyngeal tumors compared with other HNSCCs [100]. The link with the Epstein–Barr virus, similar to HPV in OPSCC, could also conceivably impact ADC values. Rasmussen et al. identified a significant correlation using the partial correlation metric and incorporating a random offset; our forest plot analyses did not yield the same level of significance as we could not correct for the offset [60]. For tumor cell count, only one of three reporting articles described significant correlations [60,61,63]. Yet a connection between cellularity and ADC metrics aligns with DWI characteristics. As data are limited, more extensive research is needed to understand these findings.

The EGFR plays a role in endothelial cell proliferation and can be targeted with Cetuximab. It has gained interest due to its link to high mutational burden [8,93,97]. However, no associations have been found between EGFR and DWI parameters such as *ADC_mean_*. There have been, however, associations observed between EGFR and DCE parameters, although with contradicting results. It was proposed that such links could possibly be based on the involvement of the EGFR in the promotion of angiogenesis through the stimulation of the production of angiogenic cytokines such as VEGF [84]. Often linked to increased tumor vascularization and aggressiveness, *K^trans^* represents the constant transfer from blood plasma to extravascular extracellular space (EES), while *K_ep_* measures the backflux exchange rate of EES to blood plasma with gadolinium contrast [101]. The presence of necrotic areas may result in lower *K^trans^* and *K_ep_* values [11] and different necrotic profiles and locations could potentially explain the divergent reported results. Higher *K^trans^* and *K_ep_* with higher EGFR values were seen in the less necrotic NSCC by Huang et al. [40], while Choi et al. [30] reported lower *K^trans^* and *K_ep_* values in high-expressing EGFR but large OPSCC that are more likely to have more necrotic areas. Studies focusing on mixed HNSCC groups [48,60] showed no significant effect, calling for further research to be categorized based on anatomical subsites.

In summary, low ADC values reflected high proliferation markers Ki-67 and, to some extent, tumor cell count; this result may have clinical implications if it could accurately guide clinicians toward more appropriate and, if required, aggressive treatment regimens. No consistent results, however, were found for associations between imaging features and the EGFR.

#### The p53 Pathway

The TP53 gene product plays a crucial role in regulating the cell cycle and trig-gering apoptosis in response to irreparable DNA damage, making it an attractive tar-get for non-invasive patient selection and treatment monitoring [98]. It should be not-ed that virtually all pharyngeal and laryngeal HNSCC tumors are affected in their p53 pathway, either through HPV E7/8 expression or in HPV-negative tumors due to TP53 gene mutations [94]. Given its degradation by HPV, TP53 mutations are absent or not biologically significant in HPV+. In addition, p53 pathway status classification (i.e., in HPV^−^) can differ across the different studies. Both high percentages of p53 expression and a complete absence of p53 expression in tumor tissue have been found to be asso-ciated with p53 pathway aberrations and TP53 mutations [98]. Together this stresses the need for an HPV and p53 status subgroup analysis that is, however, lacking or in-sufficiently statistically powered within the included studies. With this in mind, some of the included articles elude to a correlation between ADC parameters like ADCmaximum and ADCmean and p53. However, this correlation probably arises from HPV-induced cellular variations observed within the mixed cohorts [32,47,60].

### 4.3. Tumor Angiogenesis Markers: HIF-1α, VEGF, and MVD

High HIF-1α expression is induced in response to tissue hypoxia [102]. While the HIF1 pathway can have multiple cellular effects that also promote angiogenesis, most studies have not been able to support a simple and unifactorial relationship between increased hypoxia, HIF1 alpha expression, and angiogenesis or tumor growth [103]. Nonetheless, Chen et al. reported a link between the presence of MRI-determined necrotic areas in tumors and a high HIF-1α value [31]. As within hypoxic areas, the effectivity of systematic and radiotherapeutic treatment decreases; therefore, MRI monitoring of hypoxia can have a prominent role in treatment evaluation and follow-up [104]. DCE-imaging can be very useful in depicting the vascularization of tissue as *K^trans^* has notably been associated with hypoxic fractions in several other tissue types [11,105]. The lack of significant associations between the DCE parameters obtained from pharmacokinetic models and HIF-1α in the studies of this review may be attributable to the focus on nasopharyngeal SCC in two of the articles [39,40]. Tumors at this subsite differ biologically and may induce HIF-1α through alternative pathways unrelated to hypoxia [40]. 

High tumor blood flow (TBF) and blood velocity (|ⴎ|) perfusion parameters were significantly associated with high IHC HIF-1α values [40]. Similarly, increased values of time–signal intensity curve perfusion parameters (RE, ME, and MRE) also showed a significant correlation with high HIF-1α expression [42]. While hypoxia is a consequence of limited tissue blood flow and permeability, the authors of these studies speculated this correlation to be based on potential angiogenetic effects of HIF-1 in other areas. However, due to the use of different measurement methods in the articles, it is challenging to gauge the true effect.

Similar to HIF-1α, VEGF can be induced in response to tumor hypoxia through the HIF pathway or directly secreted by tumor cells [102]. Higher VEGF expression is expected in situations of low perfusion (hypoxia) or high blood flow (angiogenic tumor growth) [33,106]. This contrast in terms of MRI-based vascular profile may hamper the DCE analyses and align with the overall lack of correlations with mean values of *K^trans^*, *K_ep_*, and *V_e_* [33,39,60]. A higher blood plasma volume could be linked to more angiogenic tumor growth as described by Hu et al. and Donaldson et al. However, differences in models and VEGF assessment methods (IHC [39] and PCR [33]) make direct comparisons challenging [33,39]. 

The analysis of MVD was complicated by the use of different MVD determination methods and the lack of consistency among studies using identical MVD parameters, potentially obscuring the results. Only the contrast index (CI) perfusion parameter for the maximum gradient of the upslope phase of the enhancement curve (CI gain max) was significantly positively correlated to MVD in both reporting studies using either CD31 [66] or CD34 [68]. This suggests that the simple contrast index curve, which considers only the speed of uptake, may provide a better depiction of the number of microvessels compared with the pharmacokinetic DCE parameters.

In summary, this review presents mixed results with respect to VEGF but reveals associations between HIF-1α and several increased tissue blood flow and permeability perfusion parameters. 

### 4.4. Tumor Heterogeneity and Radiogenomics

As a non-invasive, 3D visualizing modality, medical imaging has some considerable advantages over biopsy-sampling-based techniques. Spatial and temporal intratumor heterogeneity has been shown to be extensive in multi-regional sampling studies, and has been identified as a major cause of treatment resistance and might be related to DNA repair deficits [107,108]. Unlike biopsy-based subtyping, imaging may better capture this heterogeneity, advancing future targeted treatment regimens and driving interest in such advances. In this review, both articles, however, were small in case numbers and primarily developed radiomics-based patient outcome prediction models. Thus, strongly outcome-biased, the articles subsequently investigated the biological differences. While this can help to find MRI feature/biology links, such approaches require an independent assessment of the potentially present associations with MRI features [76,77]. 

### 4.5. Limitations

While comprehensive, this review of MRI parameter associations with biological features is limited by the large variety of methods used in the included articles for calculating biological and MRI parameters. Some studies report on histogram parameters based just on the lowest or highest voxel (e.g., *ADC_minimum_* or *ADC_maximum_*), posing challenges to reproducibility. Additionally, variations in segmentation methods for tumor delineation (e.g., inclusion/exclusion of necrotic and cystic areas) impact overall measurements. Furthermore, only a modest amount of data are available for certain biological factors such as p53 and MVD, making it difficult to draw firm conclusions and highlighting the need for further research.

It is also crucial to note that biological processes do not occur in isolation. Cell proliferation and angiogenesis are intertwined through multiple cross-linked processes. Similarly, in MRI there are interconnections between parameters such as DCE, DWI, and even T1W and T2W parameters in visualizing the tissue. Understanding these complex relationships can offer more comprehensive insights into the important tumor-specific differences, paving the way for improved clinical outcome prediction and personalized patient management in HNSCC.

## 5. Conclusions

In this comprehensive review, we analyzed the relationship between biological factors and MRI in HNSCC. Across all studies, we predominantly found that HPV^+^ tumors showed lower *ADC_mean_* (SMD: 0.82; *p* < 0.001) and *ADC_minimum_* (SMD: 0.56; *p* < 0.001) values than HPV^−^ tumors. Lower ADC values correlated with elevated Ki-67 index levels in most studies (COR: −0.37; *p* = 0.051). Perfusion parameters that depict increased blood plasma volume and flow showed some associations with HIF-1α. There is potential for radiomic models to capture biological differences in tumors. However, diverse methodologies and limited reports on certain investigated biological factors necessitate further research and larger datasets. Understanding these connections can improve clinical outcome prediction and facilitate personalized patient management in HNSCC.

## Figures and Tables

**Table 1:** Characteristics of studies focused on HPV status associations with shape, stand-alone histogram T1W and T2W parameters, or radiomic features.

Study, Year	Location Inclusion Center	Study Design		Inc. (N)	Age (mean)	Male (%)	Tumor Subside	Tumor Stage	HPV Testing		HPV^+^ (n)	HPV^−^ (n)		
**Independent shape parameters**													**Sequence**	**Method**
Driessen, 2016 [35]	Utrecht, NED	R		73	61.6	64.4	OC, OP, HP, LA	T2-T4	p16+PCR		6	67	b0	Volume
Han, 2018 [38]	Suwon, KOR	R		41	62.9 †	73.2	OC, OP	All	Hybrid cap		16	25	T1c	Volume
Kawaguchi, 2020 [43]	Gifu, JAP	R		37	61.5	81.1	NA	All	p16		3	34	T1 or T2	Diameter
Martens, 2019 [45]	Amsterdam, NED	R		89 ⁑	64.6	75.2	OC, OP, HP, LA	All	p16+PCR		33	56	T1	GTV
Samolyk-Kogaczewska, 2020 [56]	Bialystok, POL	P		21	60 †	-	OC, OP, HP	All	p16 and p16+PCR		4	17	PET-MR	Volume, diameter
Vidiri, 2019 [69]	Rome, ITA	P		73	62.7	80.8	OP	All	p16+PCR		54	19	b800	Volume
**Stand-alone Histogram T1W and T2W parameters**													**Sequence**	**#Features**
Giannitto, 2020 [37]	Milan, ITA	R		32	60 †	81.3	OP	Tis-T4	p16+PCR		20 ‡	9 ‡	3DT1c	1286
Meyer, 2019 [49]	Leipzig, DEU	P		34	56.7	73.5	OC, OP, HP, LA, NA	All	p16		-	-	T1, T2	24
Ravanelli, 2018 [55]	Brescia, ITA	R		59	64.9	72.9	OP	T2-T4	HC2 DNA		28	31	3DT1c, T2, DWI	60
Kawaguchi, 2020 [43]	Gifu, JAP	R		37	61.5	81.1	NA	All	p16		3	34	T1, T2, DWI	5
**Radiomic models HPV**														
		**Train** **(N)**	**Test (N)**	**Age (mean)**	**Male (%)**	**Tumor Subside**	**Tumor Stage**	**HPV** **Testing**	**HPV^+^** **(n)**	**HPV^−^** **(n)**	**Modality**	**#Features**	**Total** **RQS**	**Domains: IM/** **FR/VA/PI/LE/OS**
Boot, 2023 [26]	Amsterdam, NED	249	-	61	68.7	OP	All	p16+PCR	91	158	T1	498	8	1/−2/6/3/−1/0
Bos, 2021 [27]	Amsterdam, NED	91	62	61	63	OP	All	p16+p53	76	77	3DT1c	1184	14	2/5/4/3/4/0
Bos, 2022 [28]	Amsterdam, NED	91	62	61	63	OP	All	p16+p53	76	77	3DT1c	1184	14	2/5/4/3/4/0
Li, 2023 [74]	Shanghai, CHN	116	25	58 †	85.8	OP	All	p16	78	63	T1c, T2	2092	11	2/5/3/1/4/0
Marzi, 2022 [46]	Rome, ITA	95	49	64.4	82.6	OP	All	p16+PCR	100	44	DWI, IVIM	157	14	2/5/4/3/4/0
Park, 2022 [53]	Seoul, KOR	108	47	58.3	83.9	OP	All	p16	136	19	T1c	140	10	2/5/1/2/2/0
Sohn, 2020 [58]	Seoul, KOR	43	19	59.3	85.5	OP	-	p16	52	10	3DT1c, T2	170	11	2/5/1/3/2/0
Suh, 2020 [59]	Seoul, KOR	40	20	59 †	83.3	OP	T0-T4	p16+PCR	48	12	T1(c), T2, DWI	1618	11	1/5/1/4/2/0

The table lists study patient population characteristics of all studies reporting stand-alone MRI parameter associations with HPV status, HPV status determination method, and used MRI parameter settings, as well as all radiomic models used to predict HPV and the radiomic quality score of these articles (RQS). Abbreviations: P = prospective study design; R = retrospective study design; Inc. = number of analyzed patients; Train = number of patients analyzed in the training cohort; Test = number of patients analyzed in the test cohort; #Features = number of features collected; OC = oral cavity; OP = oropharynx; HP = hypopharynx; LA = larynx; NA = nasopharynx; - = not available; Hybrid cap = hybrid capture assay kits; H2C DNA = HC2 high-risk HPV DNA test; GTV = gross tumor volume; RQS = radiomics quality score; RSQ domains = IM: image protocol and feature reproducibility; FR = feature reduction and validation; VA = biologic/clinical validation and utility; PI = performance index; LE = level of evidence; and OS = open science. Notes: † Median value; ‡ 3 patients did not have HPV status available; and ⁑ Nr of patients with DWI available.

**Table 2:** Characteristics of MRI diffusion and perfusion parameter association studies with HPV status.

Study, Year	Location Inclusion Center	Study Design	Inc. (N)	Age (mean)	Male (%)	Tumor Subside	Tumor Stage	HPV Testing	HPV^+^ (n)	HPV^−^ (n)	
**Diffusion parameters**											**b-values (s/mm^2^)**
Chan, 2016 [29]	Toronto, CAN	R	40	59.2	82.5	OP	All	p16	28	12	0, 1000
De Perrot, 2017 [34]	Geneva, CHE	R	105	64	71.4	OC, OP	All	p16+PCR	21	84	0, 1000
Driessen, 2016 [35]	Utrecht, NED	R	73	61.6	64.4	OC, OP, HP, LA	T2-T4	p16+PCR	6	67	0, 150, 800
Freihat, 2021 [36]	Pécs, HUN	R	33	61.4	69.7	OP	All	p16	16	17	0, 800, 1000
Han, 2018 [38]	Suwon, KOR	R	41	62.9 †	73.2	OC, OP	All	Hybrid cap	16	25	0, 1000
Kawaguchi, 2020 [43]	Gifu, JAP	R	37	61.5	81.1	NA	All	p16	3	34	0, 1000
Lenoir, 2022 [44]	Geneva, CHE	R	34	62.0 †	61.8	OP	All	p16+PCR	11	23	0, 50, 100, 500, 750, 1000
Martens, 2019 [45]	Amsterdam, NED	R	89 ⁑	64.6	75.2	OC, OP, HP, LA	All	p16+PCR	33	56	0, 1000
Marzi, 2022 [46]	Rome, ITA	R	95 *	65.0	80.0	OP	All	p16+PCR	67	28	0, 500, 800, IVIM
Meyer, 2018 [47]	Leipzig, DEU	P	34	56.7	73.5	OC, OP, HP, LA, NA	All	p16	-	-	0, 800
Nakahira, 2014 [52]	Saitama, JAP	R	26	66	92.3	OP	All	p16	12	14	0, 1000
Piludu, 2021 [54]	Rome, ITA	P	100	65.7	82.0	OP	T0-T4	p16+PCR	69	31	0, 25, 50, 75, 100, 150, 300, 500, 800, IVIM°
Ravanelli, 2018 [55]	Brescia, ITA	R	59	64.9	72.9	OP	T2-T4	HC2 DNA	28	31	0, 1000
Schouten, 2015 [57]	Amsterdam, NED	R	44	58.8	75.0	OP	T2-T4	p16+PCR	22	22	0, 750, 1000
Vidiri, 2019 [69]	Rome, ITA	P	73	62.7	80.8	OP	All	p16+PCR	54	19	0, 500, 800, IVIM
Wong, 2016 [70]	Londen, GBR	P	20	63 †	90.0	OP, HP	All	Unclear	12	8	50, 400, 800
**Perfusion parameters**											**Model**
Ahn, 2021 [25]	Seoul, KOR	P	58	59.5	82.8	OP	All	p16+PCR	45	13	Arterial Spin Labeling
Choi, 2016 [30]	Seoul, KOR	R	22	61.6	86.4	OC, OP	-	p16	15	7	Tofts and Brix
Han, 2018 [38]	Suwon, KOR	R	41	62.9 †	73.2	OC, OP	All	Hybrid cap	16	25	Extended Tofts
Meyer, 2019 [48]	Leipzig, DEU	P	30	57.0	73.3	OC, OP, HP, LA, NA	All	p16	20	10	Tofts and Kermode
Piludu, 2021 [54]	Rome, ITA	P	100	65.7	82.0	OP	T0-T4	p16+PCR	69	31	IVIM, Tofts, and Brix
Vidiri, 2019 [69]	Rome, ITA	P	73	62.7	80.8	OP	All	p16+PCR	54	19	IVIM

The table lists study patient population characteristics of all studies reporting MRI perfusion and diffusion parameter associations with HPV status, HPV status determination method, and used functional MRI settings and models. Abbreviations: P = prospective study design; R = retrospective study design; Inc. = number of analyzed patients; OC = oral cavity; OP = oropharynx; HP = hypopharynx; LA = larynx; NA = nasopharynx; - = not available; hybrid cap = hybrid capture assay kits; and H2C DNA = HC2 High-risk HPV DNA test. Notes: † Median value; ⁑ Nr of patients with DWI available. * Test set of radiomics study. ° b0, b500 and b800 were used for apparent diffusion coefficient (ADC) calculation.

**Table 3:** Characteristics of MRI parameter association studies with other biological endpoints.

Study, Year	Location Inclusion Center	Study Design	Inc. (N)	Age (mean)	Male (%)	Tumor Subside	Tumor Stage	Testing Method	Biological Feature	
**Diffusion parameters**										**b-values (s/mm^2^)**
Chen Y., 2023 [75]	Beijing, CHN	R	21	61.3	85.7	OC, LA	All	IHC	EGFR	0, 800
Dang, 2015 [32]	Calgary, CAN	P	16	56.0	87.5	OP	T2-T4	IHC	p53	-
Meyer, 2018 [47]	Leipzig, DEU	P	34	56.7	73.5	All HSNCC	All	IHC	p53, HIF-1α, VGEF, EGFR	0, 800
Meyer, 2019 [51]	Leipzig, DEU	R	34	56.7	73.5	All HNSCC	All	IHC	MVD (CD105)	0, 800
Rasmussen, 2020 [60]	Copenhagen, DNK	P	28	63 †	57.1	All HNSCC	All	IHC	p53, HIF-1α, VGEF, EGFR, Ki-67	0, 800
Shima, 2023 [72]	Sapporo, JPN	P	24	68 †	50	OC	All	IHC	Ki-67	0, 500, 1000, 1500, 2000, 2500, DKI
Surov, 2016 [61]	Leipzig, DEU	P	11	56.0	81.8	All HNSCC	All	IHC	Ki-67, CC	0, 800
Surov, 2018 [63]	Leipzig, DEU	P	32	56.5	75.0	OC, OP, HP, LA	All	IHC	Ki-67, CC	0, 800
Swartz, 2018 [65]	Utrecht, NED	R	20	61.4	55.0	OP	T2-T4	IHC	HIF-1α, Ki-67	0, 150, 800
Tse, 2010 [67]	Shatin, HKG	P	45	-	-	HNSCC	-	IHC	VGEF, EGFR	0, 100, 200, 300, 400, 500
Wu W., 2021 [71]	Foshan, CHN	P	36	47.3	77.8	NA	T2-T4	IHC	Ki-67	0, 10, 20, 40. 60, 100, 120, 160, 200, 400, 600, 800, 1000, IVIM
Wu Y., 2023 [73]	Kanton, CHN	R	25	58.9	64	NA	-	IHC	Ki-67	0, 1000, 2000
**Perfusion parameters**										**Model**
Chen Y., 2023 [75]	Beijing, CHN	R	21	61.3	85.7	OC, LA	All	IHC	EGFR	1compartment NOS
Choi, 2016 [30]	Seoul, KOR	R	22	61.6	86.4	OC, OP	-	IHC	EGFR	Tofts and Brix
Donaldson, 2015 [33]	Manchester, GBR	P	7	62.0	100	OC, HP, LA	All	PCR	VGEF	2CXM
Hu, 2018 [39]	Changsha, CHN	P	94	-	69.1	NA	All	IHC	HIF-1α, VGEF, MVD (CD34)	2compartment NOS
Huang, 2021 [40]	Hainan, CHN	R	87(70) *	49 ⁑	79 ⁑	NA	All	IHC	HIF-1α, EGFR, Ki-67	Extended Tofts
Karabay, 2022 [41]	Konak, TUR	R	33	61.9	81.8	OC, OP, LA	All	IHC	MVD (CD34,CD105)	Tofts
Liu, 2021 [42]	Nanchang, CHN	P	42	53.2	69.0	NA	All	IHC	HIF-1α	TIC
Meyer, 2019 [48]	Leipzig, DEU	P	30	57.0	73.3	All HNSCC	All	IHC	p53, HIF-1α, VGEF, EGFR	Tofts and Kermode
Meyer, 2019 [50]	Leipzig, DEU	R	30	57.2	76.7	All HNSCC	All	IHC	MVD (CD105)	Tofts and Kermode
Rasmussen, 2020 [60]	Copenhagen, DNK	P	28	63 †	57.1	HNSCC+LN (25%)	All	IHC	p53, VGEF, EGFR, Ki-67, CC	Tofts and Brix
Surov, 2017 [62]	Leipzig, DEU	P	16(11) ‡	57.0	87.5	HNSCC	All	IHC	Ki-67, MVD (CD31), CC	Tofts and Kermode
Surov, 2018 [64]	Leipzig, DEU	P	30	57.0	73.3	All HNSCC	All	IHC	Ki-67, CC	Tofts and Kermode
Tekiki, 2021 [66]	Okayama, JPN	P	21	64	57.1	OC	T1-T3	IHC	VGEF, MVD (CD31)	Contrast index
Unestubo, 2009 [68]	Okayama, JPN	P	28	65.9	50.0	OC	T2-T3	IHC	MVD (CD34)	Contrast index
**Stand-alone Histogram T1W and T2W parameters**										
Chen T., 2015 [31]	Taipei, TWN	R	218	51.0	87.2	OC	All	IHC	HIF-1α	
Dang, 2015 [32]	Calgary, CAN	P	16	56.0	87.5	OP	T2-T4	IHC	p53	
Meyer, 2019 [49]	Leipzig, DEU	P	34	56.7	73.5	All HNSCC	All	IHC	p53, HIF-1α, VGEF, EGFR, Ki-67, CC	
Samolyk-Kogaczewska, 2020 [56]	Białystok, POL	P	21	60 †	-	OC, OP, HP	All	IHC	Ki-67	

Grouped by MRI modality, the table lists the study patient population and parameter characteristics of all studies testing MRI parameter associations with pathological assessments of p53, HIF-1α, VEGF, EGFR, MVD, Ki-67, or tumor cell counts. Abbreviations: P = prospective study design; R = retrospective study design; Inc.= number of analyzed patients; OC = oral cavity; OP = oropharynx; HP = hypopharynx; LA = larynx; NA = nasopharynx; LN = lymph nodes; OT = other/non-HNSCC; - = not available; IHC = immunohistochemistry; PCR = polymerase chain reaction; CC = cell count; NOS = not otherwise specified; and TIC = time–signal intensity curve. Notes: † Median value; * subcohort for KI-67; ‡ included recurrent tumors, sub-analyses for primary; and ⁑ estimation over groups.

## Supplementary Materials

The following supporting information can be downloaded at: https://www.mdpi.com/article/10.3390/cancers15205077/s1. Supplementary Material S1: Material and methods; Supplementary Material S2: Results.
