# Supplementary material for: The Prediction of Biological Features Using Magnetic Resonance Imaging in Head and Neck Squamous Cell Carcinoma: A Systematic Review and Meta-Analysis
Source: Cancers (Basel). 2023 Oct 20;15(20):5077. doi: 10.3390/cancers15205077 (PMC10605807; doi:10.3390/cancers15205077)
Supplement: Supplementary file 1 [file cancers-15-05077-s001.zip › Supplementary material S1-Material and methods.pdf]

Supplementary material S1

Materials and Methods

Table of content:

Appendix S1 – Literature search structure and terms……………………………………………………………..Page 2

A1.1 – Search schematic…………………………………………………………………………………………Page 2

A1.2 – Search terms…………………………………………………………………………………………………Page 2

Appendix S2 – Full literature searches per database and total hits…………………………………………Page 5

A2.1 – Search Medline (Ovid) ……………………………………………………………………………….Page 5

A2.2 – Search Embase (Ovid) …………………………………………………………………………………Page 5

A2.3 – Search SCOPUS…………………………………………………………………………………………….Page 6

A2.4 – Initial search results…………………………………………………………………………………….Page 7

A2.5 – Update search results………………………………………………………………………………….Page 7

Appendix S3 – Overview of QUADAS-2 scoring tool……………………………………………………………….Page 8

Appendix S4 – Formulas……………………………………………………………………………………………………Page 11

Appendix S1 – Literature search structure and terms

A1.1 Search *schematic*:

1		2		3				
HNSCC	AND	MRI	AND	(GENES	OR	BIOMARKERS	OR	RADIGENOMICS)

A1.2 Search terms (Medline (Ovid))

MODULE 1

HNSCC

Carcinoma, Squamous Cell	exp
SCC	ti,ab,kf
"Squamous cell"	ti,ab,kf

AND

Head and Neck Neoplasms	exp

Cancer*	ti,ab,kf
Carcinom*	ti,ab,kf
Carcinogene*	ti,ab,kf
Malign*	ti,ab,kf
Neoplasm*	ti,ab,kf
Oncogene*	ti,ab,kf
Oncolog*	ti,ab,kf
Tumor*	ti,ab,kf
Tumour*	ti,ab,kf

AND

"Aerodigestive tract"	ti,ab,kf
"Aryepiglottic fold"	ti,ab,kf
Face	ti,ab,kf
Facial	ti,ab,kf
Gingiv*	ti,ab,kf
Glottis	ti,ab,kf
Head or neck	ti,ab,kf
UADT	ti,ab,kf
HNSCC	ti,ab,kf
Hypopharyn*	ti,ab,kf
Laryn*	ti,ab,kf
Mouth	ti,ab,kf
Nasopharyn*	ti,ab,kf
Oropharyn*	ti,ab,kf
Oral	ti,ab,kf
Palat*	ti,ab,kf
Pharyn*	ti,ab,kf
"Piriform sinus"	ti,ab,kf
Retromolar trigone	ti,ab,kf
SCCHN	ti,ab,kf
Sinonasal	ti,ab,kf
Subglott*	ti,ab,kf
Supraglot*	ti,ab,kf
Tongue	ti,ab,kf
Tonsil*	ti,ab,kf
Vallecular	ti,ab,kf

MODULE 2

MRI

Imaging*	ti,ab,kf

AND

Chemical Shift	ti,ab,kf
Diffusion Tensor Echo-Planar	ti,ab,kf
"Magnetization Transfer Contrast"	ti,ab,kf
MR	ti,ab,kf
"Spin Echo"	ti,ab,kf

Magnetic Resonance Imaging	exp
ADC	ti,ab,kf
"Apparent Diffusion Coefficient"	ti,ab,kf
"Diffusion Tractograph*"	ti,ab,kf
"Diffusion-weighted imaging"	ti,ab,kf
"Diffusion-weighted mr*"	ti,ab,kf
DWI	ti,ab,kf
"DW imaging"	ti,ab,kf
"DW MR*"	ti,ab,kf
fMRI	ti,ab,kf
"MR angiograph*"	ti,ab,kf
"Magnetic resonance"	ti,ab,kf
"Magnetic resonance angiograph*"	ti,ab,kf
"MRCP"	ti,ab,kf
MRI	ti,ab,kf
MRIs	ti,ab,kf
MRT	ti,ab,kf
"Nmr petmri"	ti,ab,kf
NMRI	ti,ab,kf
PET-MRI	ti,ab,kf
mpMRI	ti,ab,kf

MODULE 3

GENES

"Multiloc* Sequenc*"	ti,ab,kf

AND

Analyses	ti,ab,kf
Analysis	ti,ab,kf
Typing	ti,ab,kf

Transcription*	ti,ab,kf

AND

Elongat*	ti,ab,kf
Gene*	ti,ab,kf
Genetic*	ti,ab,kf
Initiat*	ti,ab,kf
Revers*	ti,ab,kf
Terminat*	ti,ab,kf

Biosynthes*	ti,ab,kf
Translat*	ti,ab,kf

AND

Genetic*	ti,ab,kf
mRNA	ti,ab,kf
Peptide*	ti,ab,kf
Protein*	ti,ab,kf
Ribosomal	ti.ab.kf

Gene Expression	exp	"HapMap"	ti,ab,kf
Genes, Neoplasm	exp	"Human Genome Project"	ti,ab,kf
Molecular Typing	exp	"Human Haplotype Map*"	ti,ab,kf
Sequence Analysis	exp	Immunogenetic*	ti,ab,kf
Cytogenetic*	ti,ab,kf	Molecular Typing	ti,ab,kf
DNA-Seq	ti,ab,kf	MPS	ti,ab,kf
Epigenetic*	ti,ab,kf	NGS	ti,ab,kf
Epigenomic*	ti,ab,kf	Proteogenomic*	ti,ab,kf
Genetic*	ti,ab,kf	Proteomic*	ti,ab,kf
Genomic*	ti,ab,kf	RNA-Seq	ti,ab,kf
"Gene Express*"	ti,ab,kf	WGA	ti,ab,kf
"Gene Ontolog*"	ti,ab,kf	WGS	ti,ab,kf

BIOMARKERS

"Programmed Cell Death 1"	ti,ab,kf
AND	
Receptor	ti,ab,kf
Protein	ti.ab.kf

Biomarkers, Tumor	exp
Cell Count	exp
CD3 Complex	exp
Epidermal Growth Factor	exp
Hypoxia-Inducible Factor 1	exp
Ki-67 Antigen	exp
Microvascular Density	exp
Papillomavirus Infections	exp
Proliferating Cell Nuclear Antigen	exp
Receptor, ErbB-2	exp
Tumor Suppressor Protein p53	exp
Vascular Endothelial Growth Factor A	exp
Biomarker*	ti,ab,kf
"Average nucleic area"	ti,ab,kf
"Cell count"	ti,ab,kf

CD31	ti,ab,kf
CD34	ti,ab,kf
"CD3 cell count"	ti,ab,kf
"Human papilloma virus*"	ti,ab,kf
HPV	ti,ab,kf
"Hypoxia-inducible factor 1*"	ti,ab,kf
HIF-1*	ti,ab,kf
EGFR	ti,ab,kf
HER2	ti,ab,kf
HER-2	ti,ab,kf
"Microvessel density"	ti,ab,kf
"Microvascular density"	ti,ab,kf
MVD	ti,ab,kf
"PD-1 Receptor"	ti,ab,kf
PCNA	ti,ab,kf
p53	ti,ab,kf
KI-67	ti,ab,kf
"Proliferating cell nuclear antigen"	ti,ab,kf
"Total nucleic area"	ti,ab,kf
"Vascular endothelial growth factor*"	ti,ab,kf
VEGF	ti,ab,kf
"Vessel area*"	ti,ab,kf

RADIOGENOMICS

" Radiogen*	ti,ab,kf

Appendix S2 – Full Literature searches per database and total hits

A2.1 Search Medline (Ovid)

**1**	((exp Carcinoma, Squamous Cell/ or "squamous cell".ti,ab,kf. or scc.ti,ab,kf.) and exp "Head and Neck Neoplasms"/) or ((hnscc or scchn or head or neck or UADT or "aerodigestive tract" or face or facial or mouth or gingiv* or palat* or tongue or laryn* or pharyn* or Hypopharyn* or Nasopharyn* or oropharyn* or tonsil* or "piriform sinus" or "aryepiglottic fold" or vallecular or "retromolar trigone" or glottis or subglott* or supraglot* or oral or sinonasal) and (neoplasm* or tumor* or tumour* or cancer* or malign* or oncolog* or carcinom* or carcinogene* or oncogene*)).ti,ab,kf.
**2**	exp Magnetic Resonance Imaging/ or ("magnetic resonance" or mri or "nmr petmri" or pet-mri or fMRI or MRIs or MRCP or NMRI or MRT or "mr angiograph*" or "magnetic resonance angiograph*" or "Diffusion Tractograph*" or mpMRI or DWI or "diffusion-weighted imaging" or "diffusion-weighted mr*" or "dw imaging" or "dw mr*" or ((mr or "Chemical Shift" or "Magnetization Transfer Contrast" or "Spin Echo" or "Diffusion Tensor" or Echo-Planar) and imaging*) or "Apparent Diffusion Coefficient" or adc).ti,ab,kf.
**3**	exp Genes, Neoplasm/ or exp Molecular Typing/ or exp Gene Expression/ or exp Sequence Analysis/ or (Genetic* or Cytogenetic* or "Gene Ontolog*" or HapMap or "Human Haplotype Map*" or "Human Genome Project" or Genomic* or Epigenomic* or Epigenetic* or Proteomic* or Proteogenomic* or Immunogenetic* or Molecular Typing or ("Multiloc* Sequenc*" and (typing or Analysis or Analyses)) or "Gene Express*" or ((Translat* or Biosynthes*) and (Genetic* or mRNA or Protein* or Ribosomal or peptide*)) or (Transcription* and (Genetic* or Gene* or Revers* or Elongat* or Initiat* or Terminat*)) or NGS or MPS or WGS or WGA or DNA-Seq or RNA-Seq).ti,ab,kf.
**4**	exp Biomarkers, Tumor/ or exp Papillomavirus Infections/ or Ki-67 Antigen/ or exp Cell Count/ or exp hypoxia-inducible factor 1/ or exp Vascular Endothelial Growth Factor A/ or Tumor Suppressor Protein p53/ or Epidermal Growth Factor/ or Microvascular Density/ or Proliferating Cell Nuclear Antigen/ or Receptor, ErbB-2/ or exp CD3 Complex/ or (Biomarker* or p53 or "human papilloma virus*" or HPV or ki-67 or "cell count" or "hypoxia-inducible factor 1*" or hif-1* or "vascular endothelial growth factor*" or vegf or egfr or p53 or "microvessel density" or "microvascular density" or mvd or cd34 or cd31 or "average nucleic area" or "total nucleic area" or pcna or "proliferating cell nuclear antigen" or her2 or her-2 or "vessel area*" or "cd3 cell count" or "PD-1 Receptor" or ("Programmed Cell Death 1" and (receptor or protein))).ti,ab,kf.
**5**	Radiogen*.ti,ab,kf.
**6**	3 or 4 or 5
**7**	1 and 2 and 6

A2.2 Search Embase (Ovid)

**#8**	#7 NOT ('conference abstract'/it OR 'conference paper'/it OR 'conference review'/it)
**#7**	#1 AND #2 AND #6
**#6**	#3 OR #4 OR #5
**#5**	'radiogenomics'/exp OR radiogen*:ti,ab,kw
**#4**	'tumor marker'/exp OR 'papillomavirus infection'/exp OR 'ki 67 antigen'/exp OR 'ki 67 antigen' OR 'cell count'/exp OR 'hypoxia inducible factor 1'/exp OR 'vasculotropin a'/exp OR 'protein p53'/exp OR 'epidermal growth factor'/exp OR 'microvascular density'/exp OR 'proliferating cell nuclear antigen gene'/exp OR 'epidermal growth factor receptor 2' OR 'cd3 antigen'/exp OR biomarker*:ti,ab,kw OR 'human papilloma virus*':ti,ab,kw OR hpv:ti,ab,kw OR 'ki 67':ti,ab,kw OR 'cell count':ti,ab,kw OR 'hypoxia-inducible factor 1*':ti,ab,kw OR 'hif 1*':ti,ab,kw OR 'vascular endothelial growth factor*':ti,ab,kw OR vegf:ti,ab,kw OR egfr:ti,ab,kw OR p53:ti,ab,kw OR 'microvessel density':ti,ab,kw OR 'microvascular density':ti,ab,kw OR mvd:ti,ab,kw OR cd34:ti,ab,kw OR cd31:ti,ab,kw OR 'average nucleic area':ti,ab,kw OR 'total nucleic area':ti,ab,kw OR pcna:ti,ab,kw OR 'proliferating cell nuclear antigen':ti,ab,kw OR her2:ti,ab,kw OR 'her 2':ti,ab,kw OR 'vessel area*':ti,ab,kw OR 'cd3 cell count':ti,ab,kw OR 'pd-1 receptor':ti,ab,kw OR ('programmed cell death 1':ti,ab,kw AND (receptor:ti,ab,kw OR protein:ti,ab,kw))
**#3**	'tumor gene'/exp OR 'molecular typing'/exp OR 'gene expression'/exp OR 'sequence analysis'/exp OR genetic*:ti,ab,kw OR cytogenetic*:ti,ab,kw OR 'gene ontolog*':ti,ab,kw OR hapmap:ti,ab,kw OR 'human haplotype map*':ti,ab,kw OR 'human genome project':ti,ab,kw OR genomic*:ti,ab,kw OR epigenomic*:ti,ab,kw OR epigenetic*:ti,ab,kw OR proteomic*:ti,ab,kw OR proteogenomic*:ti,ab,kw OR immunogenetic*:ti,ab,kw OR 'molecular typing':ti,ab,kw OR ('multilocus sequence':ti,ab,kw AND (typing:ti,ab,kw OR analysis:ti,ab,kw OR analyses:ti,ab,kw)) OR 'gene expression*':ti,ab,kw OR ((translation:ti,ab,kw OR biosynthes*:ti,ab,kw) AND (genetic*:ti,ab,kw OR mrna:ti,ab,kw OR protein:ti,ab,kw OR ribosomal:ti,ab,kw OR peptide:ti,ab,kw)) OR

	(transcription:ti,ab,kw AND (genetic:ti,ab,kw OR gene:ti,ab,kw OR reverse:ti,ab,kw OR elongation:ti,ab,kw OR initiation:ti,ab,kw OR termination:ti,ab,kw)) OR ngs:ti,ab,kw OR mps:ti,ab,kw OR wgs:ti,ab,kw OR wga:ti,ab,kw OR 'dna seq':ti,ab,kw OR 'rna seq':ti,ab,kw
**#2**	'nuclear magnetic resonance imaging'/exp OR 'magnetic resonance':ti,ab,kw OR mri:ti,ab,kw OR 'nmr petmri':ti,ab,kw OR 'pet mri':ti,ab,kw OR fmri:ti,ab,kw OR mris:ti,ab,kw OR mrcp:ti,ab,kw OR nmri:ti,ab,kw OR mrt:ti,ab,kw OR 'mr angiograph*':ti,ab,kw OR 'diffusion tractograph*':ti,ab,kw OR mpmri:ti,ab,kw OR dwi:ti,ab,kw OR 'diffusion-weighted imaging':ti,ab,kw OR 'diffusion-weighted mr*':ti,ab,kw OR 'dw imaging':ti,ab,kw OR 'dw mr*':ti,ab,kw OR ((mr:ti,ab,kw OR 'chemical shift':ti,ab,kw OR 'magnetization transfer contrast':ti,ab,kw OR 'spin echo':ti,ab,kw OR 'diffusion tensor':ti,ab,kw OR 'echo planar':ti,ab,kw) AND imaging*:ti,ab,kw) OR 'apparent diffusion coefficient':ti,ab,kw OR adc:ti,ab,kw
**#1**	'head and neck squamous cell carcinoma'/exp OR (('squamous cell' OR scc) AND 'head and neck tumor'/exp) OR ((hnscc:ti,ab,kw OR scchn:ti,ab,kw OR head:ti,ab,kw OR neck:ti,ab,kw OR uadt:ti,ab,kw OR 'aerodigestive tract':ti,ab,kw OR face:ti,ab,kw OR facial:ti,ab,kw OR mouth:ti,ab,kw OR gingiv*:ti,ab,kw OR palat*:ti,ab,kw OR tongue:ti,ab,kw OR laryn*:ti,ab,kw OR pharyn*:ti,ab,kw OR hypopharyn*:ti,ab,kw OR nasopharyn*:ti,ab,kw OR oropharyn*:ti,ab,kw OR tonsil*:ti,ab,kw OR 'piriform sinus':ti,ab,kw OR 'aryepiglottic fold':ti,ab,kw OR vallecular:ti,ab,kw OR 'retromolar trigone':ti,ab,kw OR glottis:ti,ab,kw OR subglott*:ti,ab,kw OR supraglot*:ti,ab,kw OR oral:ti,ab,kw OR sinonasal:ti,ab,kw) AND (neoplasm*:ti,ab,kw OR tumor*:ti,ab,kw OR tumour*:ti,ab,kw OR cancer*:ti,ab,kw OR malign*:ti,ab,kw OR oncolog*:ti,ab,kw OR carcinom*:ti,ab,kw OR carcinogene*:ti,ab,kw OR oncogene*:ti,ab,kw))

A2.3 Search SCOPUS

**6**	(TITLE-ABS ((("squamous cell" OR scc) AND "head and neck neoplasms") OR ((hnscc OR scchn OR head OR neck OR uadt OR "aerodigestive tract" OR face OR facial OR mouth OR gingiv* OR palat* OR tongue OR laryn* OR pharyn* OR hypopharyn* OR nasopharyn* OR oropharyn* OR tonsil* OR "piriform sinus" OR "aryepiglottic fold" OR vallecular OR "retromolar trigone" OR glottis OR subglott* OR supraglot* OR oral OR sinonasal) AND (neoplasm* OR tumor* OR tumour* OR cancer* OR malign* OR oncolog* OR carcinom* OR carcinogene* OR oncogene*)))) OR (AUTHKEY (((("squamous cell" OR scc) AND "head and neck neoplasms")) OR ((hnscc OR scchn OR head OR neck OR uadt OR "aerodigestive tract" OR face OR facial OR mouth OR gingiv* OR palat* OR tongue OR laryn* OR pharyn* OR hypopharyn* OR nasopharyn* OR oropharyn* OR tonsil* OR "piriform sinus" OR "aryepiglottic fold" OR vallecular OR "retromolar trigone" OR glottis OR subglott* OR supraglot* OR oral OR sinonasal) AND (neoplasm* OR tumor* OR tumour* OR cancer* OR malign* OR oncolog* OR carcinom* OR carcinogene* OR oncogene*)))) AND (TITLE-ABS (({magnetic resonance} OR mri OR {nmr petmri} OR {pet mri} OR fmri OR mris OR mrcp OR nmri OR mrt OR "mr angiograph*" OR "diffusion tractograph*" OR mpmri OR dwi OR {diffusion-weighted imaging} OR "diffusion-weighted mr*" OR {dw imaging} OR "dw mr*" OR ((mr OR {chemical shift} OR {magnetization transfer contrast} OR {spin echo} OR {diffusion tensor} OR {echo planar}) AND imaging*) OR {apparent diffusion coefficient} OR adc)) OR AUTHKEY (({magnetic resonance} OR mri OR {nmr petmri} OR {pet mri} OR fmri OR mris OR mrcp OR nmri OR mrt OR "mr angiograph*" OR "diffusion tractograph*" OR mpmri OR dwi OR {diffusion-weighted imaging} OR "diffusion-weighted mr*" OR {dw imaging} OR "dw mr*" OR ((mr OR {chemical shift} OR {magnetization transfer contrast} OR {spin echo} OR {diffusion tensor} OR {echo planar}) AND imaging*) OR {apparent diffusion coefficient} OR adc))) AND (TITLE-ABS ((genetic* OR cytogenetic* OR "gene ontolog*" OR hapmap OR "human haplotype map*" OR {human genome project} OR genomic* OR epigenomic* OR epigenetic* OR proteomic* OR proteogenomic* OR immunogenetic* OR {molecular typing} OR ({multilocus sequence} AND (typing OR analysis OR analyses)) OR "gene expression*" OR ((translation OR biosynthes*) AND (genetic* OR mrna OR protein OR ribosomal OR peptide)) OR (transcription AND (genetic OR gene OR reverse OR elongation OR initiation OR termination)) OR ngs OR mps OR wgs OR wga OR dna-seq OR rna-seq)) OR AUTHKEY ((genetic* OR cytogenetic* OR "gene ontolog*" OR hapmap OR "human haplotype map*" OR {human genome project} OR genomic* OR epigenomic* OR epigenetic* OR proteomic* OR proteogenomic* OR immunogenetic* OR {molecular typing} OR ({multilocus sequence} AND (typing OR analysis OR analyses)) OR "gene expression*" OR ((translation OR biosynthes*) AND (genetic* OR mrna OR protein OR ribosomal OR peptide)) OR (transcription AND (genetic OR gene OR reverse OR elongation OR initiation OR termination)) OR ngs OR mps OR wgs OR wga OR dna-seq OR rna-seq))) OR (TITLE-ABS ((biomarker* OR p53 OR "human papilloma virus*" OR hpv OR ki-67 OR {cell count} OR "hypoxia-inducible factor 1*" OR hif-1* OR "vascular endothelial growth factor*" OR vegf OR egfr OR p53 OR {microvessel density} OR {microvascular density} OR mvd OR cd34 OR cd31 OR {average nucleic area} OR {total nucleic area} OR pcna OR {proliferating cell nuclear antigen} OR her2 OR her-2 OR "vessel area*" OR {pd-1 receptor} OR ({programmed cell death 1} AND (receptor OR protein)))) OR AUTHKEY ((biomarker* OR p53 OR "human papilloma virus*" OR hpv OR ki-67 OR {cell count} OR "hypoxia-inducible factor 1*" OR hif-1* OR "vascular endothelial growth factor*" OR vegf OR egfr OR p53 OR {microvessel density} OR {microvascular density} OR mvd OR cd34 OR cd31 OR {average nucleic area} OR {total nucleic area} OR pcna OR {proliferating cell nuclear antigen} OR her2 OR her-2 OR "vessel area*" OR {pd-1 receptor} OR ({programmed cell death 1} AND (receptor OR protein))))) OR (TITLE-ABS (radiogen*) OR AUTHKEY (radiogen*))

**5**	TITLE-ABS(radiogen*) OR AUTHKEY(radiogen*)
**4**	TITLE-ABS((Biomarker* or p53 or "human papilloma virus*" or HPV or ki-67 or {cell count} or "hypoxia-inducible factor 1*" or hif-1* or "vascular endothelial growth factor*" or vegf or egfr or p53 or {microvessel density} or {microvascular density} or mvd or cd34 or cd31 or {average nucleic area} or {total nucleic area} or pcna or {proliferating cell nuclear antigen} or her2 or her-2 or "vessel area*" or {PD-1 Receptor} or ({Programmed Cell Death 1} and (receptor or protein)))) OR AUTHKEY((Biomarker* or p53 or "human papilloma virus*" or HPV or ki-67 or {cell count} or "hypoxia-inducible factor 1*" or hif-1* or "vascular endothelial growth factor*" or vegf or egfr or p53 or {microvessel density} or {microvascular density} or mvd or cd34 or cd31 or {average nucleic area} or {total nucleic area} or pcna or {proliferating cell nuclear antigen} or her2 or her-2 or "vessel area*" or {PD-1 Receptor} or ({Programmed Cell Death 1} and (receptor or protein))))
**3**	TITLE-ABS((Genetic* or Cytogenetic* or "Gene Ontolog*" or HapMap or "Human Haplotype Map*" or {Human Genome Project} or Genomic* or Epigenomic* or Epigenetic* or Proteomic* or Proteogenomic* or Immunogenetic* or {Molecular Typing} or ({Multilocus Sequence} and (typing or Analysis or Analyses)) or "Gene Expression*" or ((Translation or Biosynthes*) and (Genetic* or mRNA or Protein or Ribosomal or peptide)) or (Transcription and (Genetic or Gene or Reverse or Elongation or Initiation or Termination)) or NGS or MPS or WGS or WGA or DNA-Seq or RNA-Seq)) OR AUTHKEY((Genetic* or Cytogenetic* or "Gene Ontolog*" or HapMap or "Human Haplotype Map*" or {Human Genome Project} or Genomic* or Epigenomic* or Epigenetic* or Proteomic* or Proteogenomic* or Immunogenetic* or {Molecular Typing} or ({Multilocus Sequence} and (typing or Analysis or Analyses)) or "Gene Expression*" or ((Translation or Biosynthes*) and (Genetic* or mRNA or Protein or Ribosomal or peptide)) or (Transcription and (Genetic or Gene or Reverse or Elongation or Initiation or Termination)) or NGS or MPS or WGS or WGA or DNA-Seq or RNA-Seq))
**2**	TITLE-ABS (({magnetic resonance} OR mri OR {nmr petmri} OR {pet mri} OR fmri OR mris OR mrcp OR nmri OR mrt OR "mr angiograph*" OR "diffusion tractograph*" OR mpmri OR dwi OR {diffusion-weighted imaging} OR "diffusion-weighted mr*" OR {dw imaging} OR "dw mr*" OR ((mr OR {chemical shift} OR {magnetization transfer contrast} OR {spin echo} OR {diffusion tensor} OR {echo planar}) AND imaging*) OR {apparent diffusion coefficient} OR adc)) OR AUTHKEY (({magnetic resonance} OR mri OR {nmr petmri} OR {pet mri} OR fmri OR mris OR mrcp OR nmri OR mrt OR "mr angiograph*" OR "diffusion tractograph*" OR mpmri OR dwi OR {diffusion-weighted imaging} OR "diffusion-weighted mr*" OR {dw imaging} OR "dw mr*" OR ((mr OR {chemical shift} OR {magnetization transfer contrast} OR {spin echo} OR {diffusion tensor} OR {echo planar}) AND imaging*) OR {apparent diffusion coefficient} OR adc))
**1**	(TITLE-ABS ((({squamous cell} OR scc) AND {Head and Neck Neoplasms}) OR ((hnscc OR scchn OR head OR neck OR uadt OR {aerodigestive tract} OR face OR facial OR mouth OR gingiv* OR palat* OR tongue OR laryn* OR pharyn* OR hypopharyn* OR nasopharyn* OR oropharyn* OR tonsil* OR {piriform sinus} OR {aryepiglottic fold} OR vallecular OR {retromolar trigone} OR glottis OR subglott* OR supraglot* OR oral OR sinonasal) AND (neoplasm* OR tumor* OR tumour* OR cancer* OR malign* OR oncolog* OR carcinom* OR carcinogene* OR oncogene*)))) OR (AUTHKEY (((({squamous cell} OR scc) AND {Head and Neck Neoplasms})) OR ((hnscc OR scchn OR head OR neck OR uadt OR {aerodigestive tract} OR face OR facial OR mouth OR gingiv* OR palat* OR tongue OR laryn* OR pharyn* OR hypopharyn* OR nasopharyn* OR oropharyn* OR tonsil* OR {piriform sinus} OR {aryepiglottic fold} OR vallecular OR {retromolar trigone} OR glottis OR subglott* OR supraglot* OR oral OR sinonasal) AND (neoplasm* OR tumor* OR tumour* OR cancer* OR malign* OR oncolog* OR carcinom* OR carcinogene* OR oncogene*))))

A2.4 Initial search results (16-02-2023):

Date	**Database**	**Number of hits before deduplication**	**Number of hits after deduplication**
16-02-2023	Medline (Ovid)	1747	1528
16-02-2023	Embase	4410	3340
16-02-2023	Scopus	1328	168
	Total	7485	5036

A2.5 Update search results (28-07-2023):

Date	**Database**	**Number of hits before deduplication**	**Number of hits after deduplication**
28-07-2023	Medline (Ovid)	62	
28-07-2023	Embase	327	
28-07-2023	Scopus	87	
	Total	476	360

Appendix S3 – Overview of QUADAS-2 scoring tool

General info		
Authors		
Year	Year of publication	
Title		
**QUADAS-2 score form**		
**Phase 1: Review question**		
**Review question**	All: Can any MRI parameter or MRI model be used to reliably establish **<Biological factor researched>** in pretreated HNSCC<br>Example:<br>HPV: Can any MRI parameter or MRI model be used to reliably establish **HPV** status in untreated HNSCC	
Patients	Hospital setting, Diagnosed with HNSCC of al T stages, Blinded for (if any) prior testing, no HNC treatment prior to acquisition.<br><br>Factor specific:<br>HPV-status: HNSCC preferably of the oropharyngeal region<br>p53: Analyzed in HPV subgroups<br>Other: All HNSCC, but preferably no nasopharyngeal combined with other HNSCC	
Index test	Any pulse sequence of MRI of which features are extracted as stand-alone features or MRI feature-only based models	
Reference standard/ target condition	HPV: established preferably by p16 IHC combined with PCR or another combination of 2 methods<br>Ki-67: Outcome scale (5 or more levels) for Ki-67 reported or continuous percentage at highest nuclei area of slide<br>HIF-1A: Preference for continuous percentage<br>Cell count: All nuclei counted<br>VEGF: Preference for continuous percentage<br>EGFR: Preference for continuous percentage<br>p53: established by IHC testing both, lack of staining and overexpression	
**Phase 2: Flow diagram**		
**Flow diagram**	Performed separate from the score form	
**Phase 3: Risk of bias and application judgement**		
**Domain 1: patient selection**		
Describe method pt selection		
Was a consecutive or random sample of patients enrolled?		Yes/No/Unclear
Was a case-control design avoided?		Yes/No/Unclear
Did the study avoid inappropriate exclusions?	HPV: inappropriate if exclusion of patients with HPV-status unknown	Yes/No/Unclear
**A: Risk Of Bias: pt selection**	Could the selection of patients have introduced bias?	**RISK: LOW/HIGH/UNCLEAR**

	(Weighted (+/-): pre-treatment patient group (+3), Consecutive/random selection (+1), No case control (+1), Inappropriate exclusion (-1=with good reason, -2=no reason, -3=inappropriate reason)	
Describe included pt	(prior testing, presentation, intended use of index test and setting)	
**B: Applicability: Do pt match review question?**	Is there concern that the included patients do not match the review question?<br>(Matching as defined in phase 1)	**CONCERN: LOW/HIGH/UNCLEAR**
**Domain 2: Index test(s)**		
Describe index test	MRI sequence(s), DCE models, DWI b-values, contrast administration.	
Were the index test results interpreted without knowledge of the results of the reference standard?	Was the radiologist/other blinded for the biological factor? – especially in retrospective cohorts	Yes/No/Unclear
If a threshold was used, was it pre-specified?	Threshold can be optimized for a dataset and may give overoptimistic performance	Yes/No/Unclear
**A: Risk of bias: Index test**	Could the conduct or interpretation of the index test have introduced bias?	**RISK: LOW/HIGH/UNCLEAR**
Extra question	Did one or more experts perform segmentation? (preference for 2)	Yes/No/Unclear
Extra question	Was the entire tumor segmented or one slice used (preference for whole tumor)	Yes/No/Unclear
**B: Applicability: index test differ from review question**	Is there concern that the index test, its conduct, or interpretation differ from the review question?<br>(Matching as defined in phase 1 + extra questions)	**CONCERN: LOW/HIGH/UNCLEAR**
**Domain 3: Reference standard**		
Describe reference standard		
Is the reference standard likely to correctly classify the target condition?	Exemplary:<br>HPV: has only p16 IHC been applied or other combined with other HPV specific testing (PCR, e.d.)	Yes/No/Unclear
Were the reference standard results interpreted without knowledge of the results of the index test?		Yes/No/Unclear
**A: Risk of bias: reference standard**	Could the reference standard, its conduct, or its interpretation have introduced bias?	**RISK: LOW/HIGH/UNCLEAR**
Extra question	For: HIF-1A, VEGF, EGFR, p53	Yes/No/Unclear

	was a positive test/reference sample use to set the threshold? (Preference for continuous variable due to different thresholds applied)	
Extra question	For: HIF-1α, VEGF, EGFR, Ki-67, tumor cell count, p53<br>is IHC scoring a result of more than 1 expert evaluation? (preference for 2)	Yes/No/Unclear
**B: Applicability: target differ from review question?**	Is there concern that the target condition as defined by<br>the reference standard does not match the review<br>question?<br>(Matching as defined in phase 1 + extra questions)	**CONCERN: LOW/HIGH/UNCLEAR**
**Domain 4: Flow and timing**		
Describe pt who did not receive one of the tests/ excluded		
Describe the time interval/interventions between tests		
Was there an appropriate interval between index test(s) and reference standard?	HPV: Time between MRI acquisition and HPV testing	Yes/No/Unclear
Did all patients receive a reference standard?		Yes/No/Unclear
Did patients receive the same reference standard?		Yes/No/Unclear
Were all patients included in the analysis?	Were all included patients analyzed?	Yes/No/Unclear
**A. Risk of bias: flow and timing**	Could the patient flow have introduced bias?	**RISK: LOW/HIGH/UNCLEAR**

APPENDIX S4 – FORMULAS

1. Standardized mean difference calculated from mean values

$$SMD = \frac{\bar{X}_{groupA} - \bar{X}_{groupB}}{SD_{pooled}}$$ **Formula (1)**

$$SD_{pooled} = \sqrt{\frac{(n_{groupA} - 1)SD^2_{groupA} + (n_{groupB} - 1)SD^2_{groupB}}{(n_{groupA} + n_{groupB} - 2)}}$$ **Formula (2)**

SMD= Standardized mean difference; $\bar{X}$= Mean value; SD = Standard deviation; n = Sample size (reference 1)

2. Calculated 95% confidence interval (95%CI) of SMD

$$SD(SMD) = \sqrt{\frac{n_{groupA} + n_{groupB}}{n_{groupA} * n_{groupB}} + \frac{SMD^2}{2(n_{groupA} + n_{groupB})}}$$ **Formula (3)**

$$95\% \; lower \; bound = SMD - 1.96 * SD(SMD)$$ **Formula (4)**

$$95\% \; upper \; bound = SMD + 1.96 * SD(SMD)$$ **Formula (5)**

SD = Standard deviation; SMD= Standardized mean difference; n = number of patients (reference 1)

3. Standardize mean difference estimated from median values with range

$$T_1 = \frac{a+b-2m}{b-a}$$ **Formula (6)**

$$\bar{X}_{(w)} \approx w\left(\frac{a+b}{2}\right) + (1-w)m$$ **Formula (7)**

$$SD_{new} \approx \frac{b-a}{\xi(n)}$$ **Formula (8)**

Use these values in formulas (1) to (5)

T_1 = Skewness Test statistic under scenario S_1 as by Shi et al. (reference 2); $\bar{X}$ = Estimated Mean value; SD = Estimated Standard deviation; m = Median value; a, b = minimum and maximum of the range; w = Weighted average of the mid-range as calculated using formula presented by Luo et al. (reference3); n = Sample size; $\xi(n)$ = A function of the sample size as calculated using formulas presented by Wan et al. (reference 4)

4. Standardize mean difference estimated from median values with Inter quartile range

$$T_2 = \frac{q_1+q_3-2m}{q_3-q_1}$$ **Formula (9)**

$$\bar{X}_{(w)} \approx w\left(\frac{q_1+q_3}{2}\right) + (1-w)m$$ **Formula (10)**

$$SD_{new} \approx \frac{q_3-q_1}{\eta(n)}$$ **Formula (11)**

Use these values in formulas (1) to (5)

T_2 = Skewness Test statistic under scenario S_2 as by Shi et al. (reference 2); $\bar{X}$ = Estimated Mean value; SD = Estimated Standard deviation; m = Median value; q_1, q_3 = Inter quartile range; w = Weighted average of the mid-quartile range as calculated using the formula presented by Luo et al. (reference 3); n = Sample size; $\eta(n)$ = A function of the sample size as calculated using formulas presented by Wan et al. (reference 4)

Reference 1: https://handbook-5-1.cochrane.org/chapter_9/9_2_3_2_the_standardized_mean_difference.htm

Reference 2: Shi J, Luo D, Wan X, et al. Detecting the skewness of data from the five-number summary and its application in meta-analysis. Statistical Methods in Medical Research. 2023;0(0). doi:10.1177/09622802231172043

Reference 3: Luo D, Wan X, Liu J, Tong T. Optimally estimating the sample mean from the sample size, median, mid-range, and/or mid-quartile range. Stat Methods Med Res. 2018 Jun;27(6):1785-1805. doi: 10.1177/0962280216669183. Epub 2016 Sep 27. PMID: 27683581.

Reference 4: Wan X, Wang W, Liu J, Tong T. Estimating the sample mean and standard deviation from the sample size, median, range and/or interquartile range. BMC Med Res Methodol. 2014 Dec 19;14:135. doi: 10.1186/1471-2288-14-135. PMID: 25524443; PMCID: PMC4383202.
