# Supplementary material for: The Prediction of Biological Features Using Magnetic Resonance Imaging in Head and Neck Squamous Cell Carcinoma: A Systematic Review and Meta-Analysis
Source: Cancers (Basel). 2023 Oct 20;15(20):5077. doi: 10.3390/cancers15205077 (PMC10605807; doi:10.3390/cancers15205077)
Supplement: Supplementary file 1 [file cancers-15-05077-s001.zip › Supplementary material S2-Results.pdf]

Supplementary material S2

Results

Table of content:

Appendix S1 – Overall 'Risk of Bias' and 'Applicability' score per biological factor………………..Page 2

Appendix S2 – Individual 'Risk of Bias' and 'Applicability' score……………….……………………………..Page 3

Appendix S3 – Overview of proportion MRI parameter studies reporting significant associations with biological features.……………………………………………………………………………………………Page 4

A3.1 – Stand-alone conventional T1W and T2W imaging parameters………………………..Page 10

A3.2 – Diffusion parameters ……………………………………………………………………………………Page 4

A3.2 – Perfusion parameters……………………………………………………………………………………Page 7

Appendix S4 – Overview of proportion MRI parameter studies reporting significant associations with biological features.……………………………………………………………………………………………Page 12

A4.1 – Stand-alone conventional T1W and T2W imaging parameters………………………..Page 13

A4.2 – Diffusion parameters ……………………………………………………………………………………Page 12

A4.2 – Perfusion parameters……………………………………………………………………………………Page 12

APPENDIX S1 – OVERALL RISK OF BIAS AND APPLICABILITY SCORE PER BIOLOGICAL FACTOR

Figure A1.1 'Risk of Bias' and 'Applicability' assessment sorted by biological factor. Values indicate the number of studies within the respective QUADAS-2 'risk of bias' or 'applicability' class. Green or "low" indicates a low risk of bias in the study and/or low concern regarding applicability. Yellow or "unclear/medium" suggests either insufficient information provided or non-ideal study characteristics influencing applicability or risk of bias. Red or "high" signifies significant concerns regarding bias or applicability.

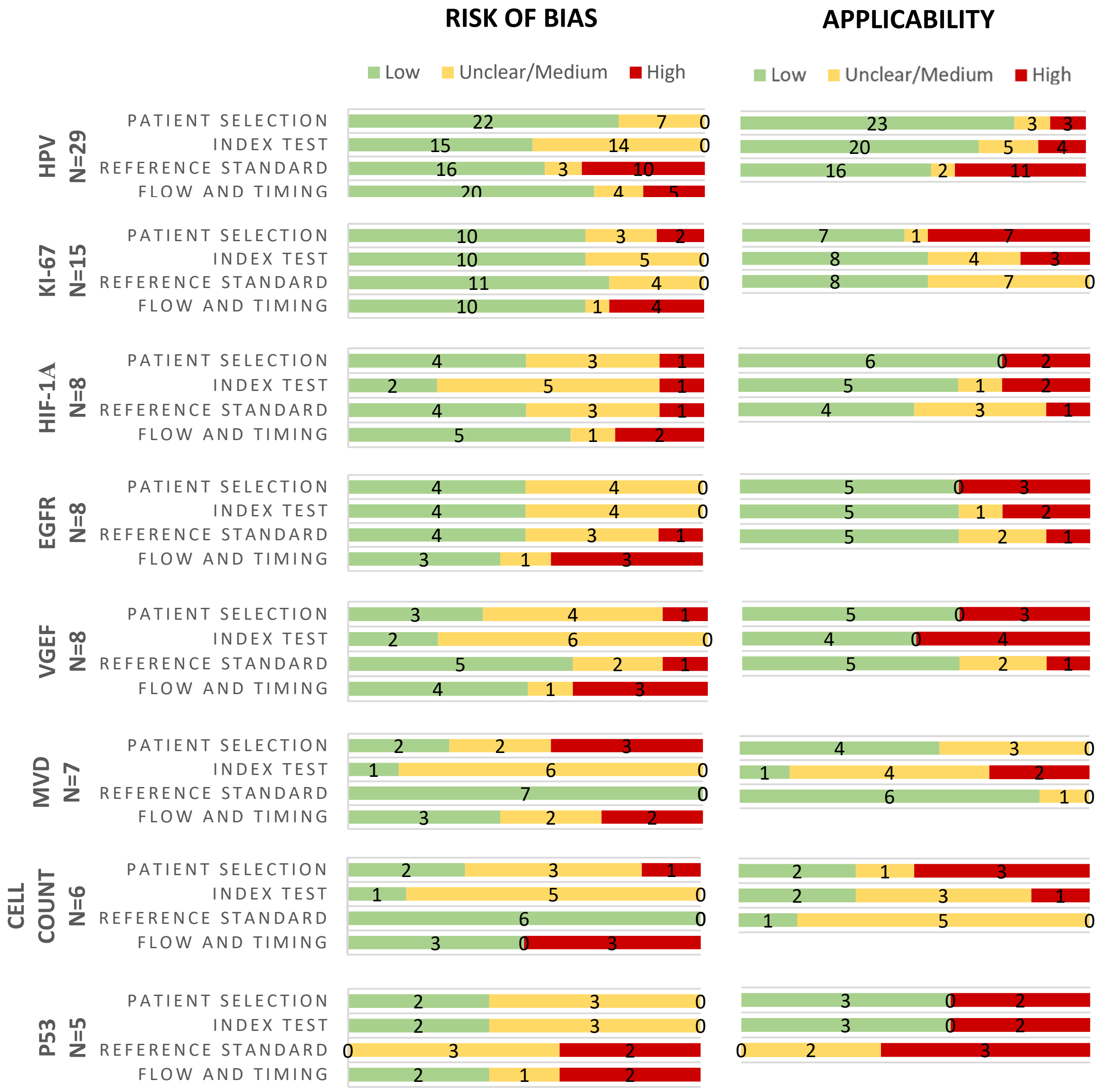

APPENDIX S2 – INDIVIDUAL QUADAS-2 'RISK OF BIAS' AND 'APPLICABILITY' SCORES PER INCLUDED STUDY

Table A2.1 'Risk of Bias' and 'Applicability' assessment for each independent study. Values indicate the numbers of studies within the respective QUADAS-2 'risk of bias' or 'applicability' class. Green or "low" indicates a low risk of bias in the study and/or low concern regarding applicability. Yellow or "unclear/medium" suggests either insufficient information provided or non-ideal study characteristics influencing applicability or risk of bias. Red or "high" signifies significant concerns regarding bias or applicability.

		Risk of Bias				Applicability		
**Author, year**	**Biological factors**	Patient Selection	Index test	Reference standard	Flow and timing	Patient selection	Index test	Reference standard
Ahn, 2021	HPV	Low	Low	Low	Low	Low	Low	Low
Boot, 2023	HPV	Unclear	Unclear	Low	High	Low	Low	Low
Bos, 2021	HPV	Low	Low	Low	Unclear	Low	Low	Low
Bos, 2022	HPV	Low	Low	Low	Unclear	Low	Low	Low
Chan, 2016	HPV	Low	Low	High	High	Low	High	High
Chen T., 2015	HIF-1α	High	High	Low	Low	High	High	Low
Chen Y., 2023	EGFR	Low	Low	Low	Low	Low	Unclear	Low
Choi, 2016	HPV	Low	Unclear	High	Low	Low	Low	High
Dang, 2015	P53	Low	Low	High	Low	Low	High	High
De Perrot, 2017	HPV	Low	Low	Low	Low	Low	Low	Low
Donaldson, 2015	VGEF	Low	Unclear	Low	Low	Low	Low	Low
Driessen, 2016	HPV	Low	Unclear	Low	Low	Low	Low	Low
Freihat, 2021	HPV	Low	Low	High	Low	Low	High	High
Gao, 2021	Whole trancriptomic sequencing	Low	Low	Low	Unclear	Unclear	Low	Unclear
Giannitto, 2020	HPV	Low	Unclear	Low	Low	Low	Low	Low
Han, 2018	HPV	Low	Low	Low	Low	Low	Low	Low
Hu, 2018	HIF-1α, VGEF, MVD (CD34)	Low	Unclear	Low	Low	Low	High	Low
Huang, 2021	HIF-1α, EGFR, KI-67	Low	Low	Unclear	Low	Low	Low	Low
Karabay, 2022	MVD (CD34,CD105)	Low	Low	Low	Low	Low	Low	Low
Kawaguchi, 2020	HPV	Unclear	Low	High	Low	Unclear	Unclear	High
Lenoir, 2022	HPV	Low	Low	Low	Low	Low	Unclear	Low
Li, 2023	HPV	Unclear	Low	High	Low	Low	Low	High
Liu, 2021	HIF-1α	Low	Unclear	Low	Low	Low	Unclear	Unclear
Martens, 2019	HPV	Low	Low	Unclear	Low	Unclear	Low	Unclear
Marzi, 2022	HPV	Low	Unclear	Low	High	Low	Unclear	Low
Meyer, 2018 (A)	HPV, P53, HIF-1α, VGEF, EGFR	Unclear	Unclear	High	High	High	Low	High
Meyer, 2019 (B)	HPV, P53, HIF-1α, VGEF, EGFR	Unclear	Unclear	High	Unclear	Low	Low	High
Meyer,2019(C)	HPV, P53, HIF-1α, VGEF, EGFR, KI-67, CC	Unclear	Unclear	High	High	High	Low	High
Meyer, 2019 (D)	MVD (CD105)	Unclear	Unclear	Low	Unclear	Unclear	Unclear	Low
Meyer, 2019 (E)	MVD (CD105)	Unclear	Unclear	Low	Unclear	Unclear	Unclear	Low

Nakahira, 2014	HPV	green	green	yellow	green		green	red	red
Park, 2022	HPV	green	yellow	red	green		green	green	red
Piludu, 2021	HPV	green	yellow	green	green		green	yellow	green
Rasmussen, 2020	VGEF, EGFR, KI-67, CC	green	green	green	green		red	red	green
Rasmussen, 2020	P53	green	green	yellow	green		red	red	red
Ravanelli, 2018	HPV	green	green	green	green		green	green	green
Samolyk-Kogaczewska, 2020	HPV, KI-67	green	green	green	yellow		green	red	red
Schouten, 2015	HPV	green	green	green	green		green	yellow	green
Shima, 2023		green	green	yellow	green		green	red	green
Sohn, 2020	HPV	yellow	yellow	red	green		green	green	red
Suh, 2020	HPV	green	yellow	green	green		green	green	green
Surov, 2016 (A)	KI-67, CC	yellow	yellow	green	red		red	green	yellow
Surov, 2017 (B)	KI-67, MVD (CD31), CC	red	yellow	green	red		yellow	yellow	yellow
Surov, 2018 (C)	KI-67, CC	green	yellow	green	green		green	yellow	yellow
Surov, 2018 (D)	KI-67, CC	yellow	yellow	green	green		red	yellow	yellow
Swartz, 2018	HIF-1α , KI-67	green	green	green	green		green	green	green
Tekiki, 2021	VGEF, MVD (CD31)	red	yellow	green	green		green	red	green
Tse, 2010	VGEF, EGFR	yellow	green	green	red		red	red	green
Unestubo, 2009	MVD (CD34)	red	yellow	green	red		green	yellow	green
Vidiri, 2019	HPV	green	yellow	green	green		green	green	green
Wong, 2016	HPV	green	yellow	yellow	green		yellow	green	yellow
Wu W., 2021	KI-67	green	green	green	green		green	green	green
Wu, Y. 2023		red	green	yellow	yellow		yellow	yellow	yellow
Zhang, 2020	Genes panel sequencing	green	green	green	yellow		yellow	green	yellow

APPENDIX S3 – OVERVIEW OF PROPORTION MRI PARAMETER STUDIES REPORTING SIGNIFICANT ASSOCIATIONS WITH BIOLOGICAL FEATURES.

Tables depicting the frequency of reported significant correlations between the stated biological factors and the individual MRI parameter out of the total number of correlations of the researching studies, displayed as nr.Significant[significant references]/nr.Total[Other reporting references]. If the percentage of significant correlations exceeds 50%, the corresponding numbers are presented in bold font. The tables are sorted by MRI sequence technique: (A3.1) Diffusion parameters (including all IVIM and DKI parameters), (A3.2) Perfusion parameters (including pharmacokinetic models, time-signal intensity curve (TIC), and arterial spin labeling (ASL) parameters), and (A3.3) stand-alone conventional t1w and t2w imaging parameters.

A3.1 STAND-ALONE CONVENTIONAL T1W AND T2W IMAGING PARAMETERS

		**Conventional T1W(+c), T2W, DWI or not specified**								**DWI**	**T1W**		**T2W**	
**BIOLOGICAL FACTOR**		Volume		Diameter		Necrosis on MRI				SIR	SIR	HSL	SIR	LSL
HPV	All	0/5[A,B,D,E**,F]		0/1[E**]		-				0/1[C]	0/1[C]	-	0/1[C]	-
	P16	0/1[E]		0/1[E]		-				0/1[C]	0/1[C]	-	0/1[C]	-
	P16+PCR	0/4[A,D,E,F]		0/1[E]		-				-	-	-	-	-
	Other	0/1[B]			-	-				-	-	-	-	-
Ki-67		0/1[E]		0/1[E]		-				-	-	0/1[I]	-	0/1[I]
HIF-1α		-		-		1/1[I]				-	-	-	-	-
		**Conventional T1W(+c)**												
		Mean	Min	Max	Median	Mode	SD	Kurtosis	Skewness	Entropy	P10	P25	P75	P90
HPV	All	0/2[G,H]	0/1[G]	0/1[G]	0/1[G]	0/1[G]	0/1[G]	0/1[G]	0/1[G]	0/1[G]	0/1[G]	0/1[G]	0/1[G]	0/1[G]
	P16	0/1[G]	0/1[G]	0/1[G]	0/1[G]	0/1[G]	0/1[G]	0/1[G]	0/1[G]	0/1[G]	0/1[G]	0/1[G]	0/1[G]	0/1[G]
	Other	0/1[H]	-	-	-	-	0/1[H]	1/1[H]	0/1[H]	0/1[H]	-	-	-	-
Ki-67		0/1[G]	0/1[G]	0/1[G]	0/1[G]	0/1[G]	0/1[G]	0/1[G]	0/1[G]	0/1[G]	0/1[G]	0/1[G]	0/1[G]	0/1[G]
HIF-1α		0/1[G]	0/1[G]	0/1[G]	0/1[G]	0/1[G]	0/1[G]	0/1[G]	0/1[G]	0/1[G]	0/1[G]	0/1[G]	0/1[G]	0/1[G]
EGFR		0/1[G]	0/1[G]	0/1[G]	0/1[G]	0/1[G]	0/1[G]	0/1[G]	0/1[G]	0/1[G]	0/1[G]	0/1[G]	0/1[G]	0/1[G]
VGEF		0/1[G*]	0/1[G]	0/1[G]	0/1[G*]	0/1[G*]	0/1[G]	0/1[G]	0/1[G]	0/1[G]	1/1[G*]	0/1[G*]	0/1[G]	0/1[G]
Tumor cell count		0/1[G]	0/1[G]	0/1[G]	0/1[G]	0/1[G]	0/1[G]	0/1[G]	0/1[G]	0/1[G]	0/1[G]	0/1[G]	0/1[G]	0/1[G]
p53		0/1[G]	0/1[G]	0/1[G]	0/1[G]	0/1[G]	0/1[G]	0/1[G]	0/1[G]	1/1[G*]	0/1[G]	0/1[G]	0/1[G]	0/1[G]
		**Conventional T2W**												
		Mean	Min	Max	Median	Mode	SD	Kurtosis	Skewness	Entropy	P10	P25	P75	P90
HPV	All	1[H]/2[G]	0/1[G]	0/1[G]	0/1[G]	0/1[G]	0/1[G]	0/1[G]	0/1[G]	0/1[G]	0/1[G]	0/1[G]	0/1[G]	0/1[G]
	P16	0/1[G]	0/1[G]	0/1[G]	0/1[G]	0/1[G]	0/1[G]	0/1[G]	0/1[G]	0/1[G]	0/1[G]	0/1[G]	0/1[G]	0/1[G]

	Other	1/1[H]	-	-	-	-	0/1[H]	0/1[H]	0/1[H]	0/1[H]	-	-	-	-
Ki-67		0/1[G*]	0/1[G]	0/1[G]	0/1[G*]	0/1[G*]	0/1[G]	0/1[G]	0/1[G]	0/1[G]	0/1[G]	0/1[G]	0/1[G*]	0/1[G*]
HIF-1α		0/1[G]	0/1[G]	0/1[G]	0/1[G]	0/1[G]	0/1[G]	0/1[G]	0/1[G]	0/1[G]	0/1[G†]	0/1[G]	0/1[G]	0/1[G†]
EGFR		0/1[G]	0/1[G]	0/1[G]	0/1[G]	0/1[G]	0/1[G]	0/1[G]	0/1[G]	0/1[G]	0/1[G]	0/1[G]	0/1[G]	0/1[G]
VGEF		0/1[G]	0/1[G]	0/1[G]	0/1[G]	0/1[G]	0/1[G]	0/1[G]	0/1[G]	0/1[G*]	0/1[G]	0/1[G]	0/1[G]	0/1[G]
Tumor cell count		0/1[G]	0/1[G]	0/1[G]	0/1[G]	0/1[G]	0/1[G]	0/1[G*]	0/1[G]	0/1[G]	0/1[G]	0/1[G]	0/1[G]	0/1[G]
p53		0/1[G*]	0/1[G]	0/1[G†]	0/1[G*]	0/1[G*]	0/1[G†]	0/1[G]	0/1[G]	0/1[G]	0/1[G]	0/1[G*]	0/1[G*]	0/1[G†]

Table shows the proportion of studies (number significant / number not significant) reporting significant associations between different functional MRI parameters and biological features as indicated. Proportions of more than half testing the indicated association are highlighted in bold.

Abbreviations = Min: minimum; max: maximum; SD: Standard deviation; P10,P25,P75,P90: percentile 10, 25, 75, 90; SIR: signal intensity ratio; HSL: High signal lesion; LSL: low signal lesion; SD: standard deviation; P10/25/75/90: percentile

*Note: *Significant for P16 positive subgroup analyses; †significant in P16 negative subgroup analyses; ⁑Volume defined by CT or PET/MRI*

a. Driessen, 2016 (article reference: [35])
b. Han, 2018 (article reference: [38])
c. Kawaguchi, 2020 (article reference: [43])
d. Martens, 2019 (article reference: [45])
e. Samolyk-Kogaczewska, 2020 (article reference: [56])
f. Vidiri, 2019 (article reference: [69])
g. Meyer, 2019 (article reference: [49])
h. Ravanelli, 2018 (article reference: [55])
i. Chen T., 2015 (article reference: [31])

A3.2 Diffusion parameters

		**Apparent diffusion coefficient (ADC) (10^{-3} mm^2/s)**												
Biological factor		Mean	Min	Max	Median	Mode	SD	Kurtosis	Skewness	Entropy	P10	P25	P75	P90
HPV	All	**10[A-D,G,H,J-L,N] /15[E,F,I,M,O]**	3[F,H,J] /7[B,E,I,M]	**2[B,H]/3[I]**	1[B]/2[I]	0/1[I]	1[H]/2[L]	2[B,G]/5[H,I,L]	2[B,G]/5[H,I,L]	0/2[I,L]	0/1[I]	1[B]/2[I]	1[B]/2[I]	0/1[I]
	P16	**3[A,D,J]/5[F,I]**	**2[F,J]/3[I]**	0/1[I]	0/1[I]	0/1[I]	-	0/1[I]	0/1[I]	0/1[I]	0/1[I]	0/1[I]	0/1[I]	0/1[I]
	PCR+P16	**6[B,C,G,H,K,N]/7[M]**	1[H]/3[B,M]	**2/2[B,H]**	**1/1[B]**	-	**1/1[H]**	**2[B,G]/3[H]**	**2[B,G]/3[H]**	-	-	**1/1[B]**	**1/1[B]**	-
	Other	1[L]/3[E,O]	0/1[E]	-	-	-	0/1[L]	0/1[L]	0/1[L]	0/1[L]	-	-	-	-
Ki-67		**5[Q-T,X]/6[U]**	1[S]/3[R,U]	2[R,S]/2	**1/1[S]**	**1/1[S]**	0/1[S]	0/1[S]	0/1[S]	0/1[S]	**1/1[S]**	**1/1[S]**	**1/1[S]**	**1/1[S]**
HIF-1α		0/2[T,I]	0/1[I]	0/1[I]	0/1[I]	0/1[I]	0/1[I]	0/1[I]	0/1[I]	0/1[I*]	0/1[I]	0/1[I*]	0/1[I]	0/1[I*]
EGFR		0/4[Q,I,V,Z]	0/1[I]	0/1[I]	0/1[I]	0/1[I]	0/1[I]	0/1[I]	0/1[I]	0/1[I]	0/1[I]	0/1[I]	0/1[I]	0/1[I]
VGEF		0/2[Q,I]	0/1[I]	0/1[I]	0/1[I]	0/1[I†]	0/1[I]	0/1[I]	0/1[I]	0/1[I]	0/1[I]	0/1[I]	0/1[I]	0/1[I]

MVD	All	0/1[W]	0/1[W]	0/1[W]	0/1[W]	0/1[W]	0/1[W]	0/1[W]	0/1[W]	0/1[W]	0/1[W]	0/1[W]	0/1[W]	0/1[W]
	CD105	0/1[W]	0/1[W]	0/1[W]	0/1[W]	0/1[W]	0/1[W]	0/1[W]	0/1[W]	0/1[W]	0/1[W]	0/1[W]	0/1[W]	0/1[W]
Tumor cell count		1[S]/3[Q,R]	1[S]/2[R]	0/2[R,S]	**1/1[S]**	**1/1[S]**	0/1[S]	**1/1[S]**	0/1[S]	0/1[S]	**1/1[S]**	**1/1[S]**	**1/1[S]**	**1/1[S]**
p53		1[Q]/2[I]	0/1[I]	**1/1[I†]**	0/1[I]	0/1[I]	0/1[I]	0/1[I*]	0/1[I]	0/1[I]	0/1[I]	0/1[I]	0/1[I†]	0/1[I†]
		**ADC (10^{-3} мм²/s)**			**Intravoxel incoherent motion (IVIM) (10^{-3} мм²/s)**									
		IQR	P10-P90	GTV	D_t	ƒ(%)	D*	D* x ƒ	D_t min	D* min	ƒ min	D_t max	D* max	ƒ max
HPV	All	**1/1[B]**	**1/1[H]**	0/1[H]	**3/3[K,N,P]**	0/1[N]	0/1[N]	0/1[N]	-	-	-	-	-	-
	PCR+P16	**1/1[B]**	**1/1[H]**	0/1[H]	**3/3[K,N,P]**	0/1[N]	0/1[N]	0/1[N]	-	-	-	-	-	-
Ki-67		-	-	-	0/1[U]	0/1[U]	0/1[U]	-	0/1[U]	0/1[U]	0/1[U]	-	-	-
		**Diffusion Kurtosis imaging (DKI) (10^{-3} mm²/s)**												
		K	D_k	DDC	α	D_{slow}	MK	Ka	Kr	FAk	MD	Da	Dr	FA
Ki-67		**1/1[X]**	**1/1[X]**	**1/1[X]**	0/1[X]	**1/1[X]**	**1/1[Y]**	**1/1[Y]**	**1/1[Y]**	0/1[Y]	**1/1[Y]**	**1/1[Y]**	**1/1[Y]**	0/1[Y]

Table shows the proportion of studies (number significant / number not significant) reporting significant associations between different functional MRI parameters and biological features as indicated. Proportions of more than half testing the indicated association are highlighted in bold.

Abbreviations: Min: minimum; max: maximum; SD: Standard deviation; P10,P25,P75,P90: percentile 10, 25, 75, 90; IQR: interquartile range; P10-P90: ADC between percentile 10 and 90; GTV: gross tumor volume; D_t: tissue diffusion coefficient; f: perfusion fraction; D: perfusion related diffusion coefficient; K: kurtosis value; D_K: the kurtosis-corrected diffusion coefficient; DDC: distributed diffusion coefficient; α: diffusion heterogeneity, D_{slow}: slow diffusion coefficient; MK: mean kurtosis; Ka: axial kurtosis; Kr: radial kurtosis; FAk: fractional anisotropy of kurtosis; MD: mean diffusivity; DA: axial diffusivity; Dr: radial diffusivity; FA: fractional anisotropy*
*Notes: *Significant for P16 positive subgroup analyses; †significant in P16 negative subgroup analyses*

a. Chan, 2016 (article reference: [29])
b. De Perrot, 2017 (article reference: [34])
c. Driessen, 2016 (article reference: [35])
d. Freihat, 2021 (article reference: [36])
e. Han, 2018 (article reference: [38])
f. Kawaguchi, 2020 (article reference: [43])
g. Lenoir, 2022 (article reference: [44])
h. Martens, 2019 (article reference: [45])
i. Meyer, 2018 (article reference: [47])
j. Nakahira, 2014 (article reference: [52])
k. Piludu, 2021 (article reference: [54])
l. Ravanelli, 2018 (article reference: [55])
m. Schouten, 2015 (article reference: [57])
n. Vidiri, 2019 (article reference: [69])
o. Wong, 2016 (article reference: [70])
p. Marzi, 2022 (article reference: [46])
q. Rasmussen, 2020 (article reference: [60])
r. Surov, 2016 (article reference: [61])

s. Surov, 2018 (article reference: [63])
t. Swartz, 2018 (article reference: [65])
u. Wu W., 2021 (article reference: [71])
v. Tse, 2010 (article reference: [67])
w. Meyer, 2019 (article reference: [51])
x. Shima, 2023 (article reference: [72])
y. Wu Y., 2023 (article reference: [73])
z. Chen Y., 2023 (article reference: [75])

A3.2 Perfusion parameters

		Volume transfer constant (K^{trans})												
**Biological factor**		Mean	Min	Max	Median	Mode	SD	Kurtosis	Skewness	Entropy	P10	P25	P75	P90
HPV	All	1[B]/4[C-E]	0/1[D]	0/1[D]	1[B]/2[D]	0/1[D]	0/1[D]	0/2[B,D]	0/2[B,D]	0/1[D]	0/1[D]	1[B]/2[D]	1[B]/2[D]	0/1[D]
	P16	1[B]/2[D]	0/1[D]	0/1[D]	1[B]/2[D]	0/1[D]	0/1[D]	0/2[B,D]	0/2[B,D]	0/1[D]	0/1[D]	1[B]/2[D]	1[B]/2[D]	0/1[D]
	PCR+P16	0/1[E]	-	-	-	-	-	-	-	-	-	-	-	-
	Other	0/1[C]	-	-	-	-	-	-	-	-	-	-	-	-
Ki-67		1[I]/4[F,H,J]	**1/1[J]**	0/1[J]	0/1[J]	0/1[J]	0/1[J]	0/1[J]	**1/1[J]**	0/1[J]	**1/1[J]**	0/1[J]	0/1[J]	0/1[J]
HIF-1α		0/3[D,F,K]	0/1[D]	0/1[D]	0/1[D]	0/1[D]	0/1[D]	0/1[D*]	0/1[D]	0/1[D]	0/1[D]	0/1[D]	0/1[D]	0/1[D]
EGFR		2[B,F]/5[D,H,Q]	0/1[D]	0/1[D]	0/2[B,D]	0/1[D]	0/1[D]	0/2[B,D]	0/2[B,D]	0/1[D*]	0/1[D]	0/2[B,D]	0/2[B,D]	0/1[D]
VGEF		0/3[D,H,K]	0/1[D]	0/1[D*]	0/1[D]	0/1[D]	0/1[D]	0/1[D]	0/1[D]	0/1[D]	0/1[D]	0/1[D]	0/1[D]	0/1[D]
MVD	All	1[G⁑]/4[I,K,O]	0/1[O]	0/1[O]	0/1[O]	0/1[O]	0/1[O]	0/1[O]	0/1[O]	0/1[O]	0/1[O]	0/1[O]	0/1[O]	0/1[O]
	CD31	0/1[I]	-	-	-	-	-	-	-	-	-	-	-	-
	CD34	1[G⁑]/2[K]	-	-	-	-	-	-	-	-	-	-	-	-
	CD105	0/2[G⁑,O]	0/1[O]	0/1[O]	0/1[O]	0/1[O]	0/1[O]	0/1[O]	0/1[O]	0/1[O]	0/1[O]	0/1[O]	0/1[O]	0/1[O]
Tumor cell count		0/3[H,I,J]	0/1[J]	0/1[J]	0/1[J]	0/1[J]	0/1[J]	1/1[J]	0/1[J]	0/1[J]	0/1[J]	0/1[J]	0/1[J]	0/1[J]
p53		1[H]/2[D]	0/1[D]	0/1[D]	0/1[D]	0/1[D]	0/1[D]	0/1[D]	0/1[D]	0/1[D]	0/1[D]	0/1[D]	0/1[D]	0/1[D]

		Rate constant (K_{ep})												
		Mean	Min	Max	Median	Mode	SD	Kurtosis	Skewness	Entropy	P10	P25	P75	P90
HPV	All	0/4[B-E]	0/1[D]	0/1[D]	0/2[B,D]	0/1[D]	0/1[D]	1[D]/2[B]	0/2[B,D]	0/1[D]	0/1[D]	1[B]/2[D]	0/2[B,D]	0/1[D]
	P16	0/2[B,D]	0/1[D]	0/1[D]	0/2[B,D]	0/1[D]	0/1[D]	1[D]/2[B]	0/2[B,D]	0/1[D]	0/1[D]	1[B]/2[D]	0/2[B,D]	0/1[D]
	PCR+P16	0/1[E]	-	-	-	-	-	-	-	-	-	-	-	-
	Other	0/1[C]	-	-	-	-	-	-	-	-	-	-	-	-
Ki-67		0/3[F,I,J]	0/1[J]	0/1[J]	0/1[J]	0/1[J]	0/1[J]	0/1[J]	0/1[J]	**1/1[J]**	0/1[J]	0/1[J]	0/1[J]	0/1[J]
HIF-1α		0/3[D,F,K]	0/1[D]	0/1[D]	0/1[D]	0/1[D]	0/1[D]	0/1[D]	0/1[D]	0/1[D*]	0/1[D]	0/1[D]	0/1[D]	0/1[D]
EGFR		1[B]/4[D,F,Q]	0/1[D]	0/1[D]	1[B]/2[D]	0/1[D]	0/1[D]	0/2[B,D]	0/2[B,D]	**1/1[D]**	0/1[D]	1[B]/2[D]	1[B]/2[D]	0/1[D]
VGEF		1/2[D,K]	0/1[D]	0/1[D]	0/1[D]	0/1[D*]	0/1[D]	0/1[D]	0/1[D]	0/1[D]	0/1[D]	0/1[D]	0/1[D]	0/1[D]

MVD	All	1[O]/4[G⁑,i,k]	0/1[O]	0/1[O]	0/1[O]	**1/1[O]**	0/1[O]	0/1[O]	0/1[O]	0/1[O]	**1/1[O]**	**1/1[O]**	**1/1[O]**	0/1[O]
	CD31	0/1[I]	-	-	-	-	-	-	-	-	-	-	-	-
	CD34	0/2[G⁑,K]	-	-	-	-	-	-	-	-	-	-	-	-
	CD105	1[O]/2[G⁑]	0/1[O]	0/1[O]	0/1[O]	**1/1[O]**	0/1[O]	0/1[O]	0/1[O]	0/1[O]	1/1[O]	1/1[O]	1/1[O]	0/1[O]
Tumor cell count		0/2[I,J]	0/1[J]	0/1[J]	0/1[J]	0/1[J]	0/1[J]	0/1[J]	0/1[J]	0/1[J]	0/1[J]	0/1[J]	0/1[J]	0/1[J]
p53		0/1[D]	0/1[D]	0/1[D]	0/1[D]	0/1[D]	0/1[D]	0/1[D]	0/1[D]	0/1[D]	0/1[D]	0/1[D]	0/1[D]	0/1[D]
		**Extravascular space (V_e)**												
		Mean	Min	Max	Median	Mode	SD	Kurtosis	Skewness	Entropy	P10	P25	P75	P90
HPV	All	0/4[B-E]	0/1[D]	0/1[D]	0/2[B,D]	0/1[D]	0/1[D]	0/2[B,D]	0/2[B,D]	0/1[D]	0/1[D]	o/2[B,D]	0/2[B,D]	0/1[D]
	P16	0/2[B,D]	0/1[D]	0/1[D]	0/2[B,D]	0/1[D]	0/1[D]	0/2[B,D]	0/2[B,D]	0/1[D]	0/1[D]	0/2[B,D]	0/2[B,D]	0/1[D]
	PCR+P16	0/1[E]	-	-	-	-	-	-	-	-	-	-	-	-
	Other	0/1[C]	-	-	-	-	-	-	-	-	-	-	-	-
Ki-67		0/3[F,I,J]	**1/1[J]**	0/1[J]	0/1[J]	0/1[J]	0/1[J]	0/1[J]	0/1[J]	**1/1[J]**	0/1[J]	0/1[J]	0/1[J]	0/1[J]
HIF-1α		1/3[D,F,K]	0/1[D]	0/1[D]	0/1[D]	0/1[D]	0/1[D]	0/1[D]	0/1[D]	0/1[D]	0/1[D]	0/1[D]	0/1[D]	0/1[D]
EGFR		1/4[B,D,F,Q]	0/1[D]	0/1[D]	0/2[B,D]	0/1[D]	0/1[D]	0/2[B,D]	0/2[B,D]	0/1[D]	0/1[D†]	0/2[B,D]	0/2[B,D]	0/1[D]
VGEF		0/2[D,K]	0/1[D]	0/1[D]	0/1[D]	0/1[D]	0/1[D]	0/1[D]	0/1[D]	0/1[D]	0/1[D]	0/1[D]	0/1[D]	0/1[D]
MVD	All	0/4[G⁑,i,k,o]	0/1[O]	0/1[O]	0/1[O]	0/1[O]	0/1[O]	0/1[O]	0/1[O]	0/1[O]	0/1[O]	0/1[O]	0/1[O]	0/1[O]
	CD31	0/1[I]	-	-	-	-	-	-	-	-	-	-	-	-
	CD34	0/2[G⁑,K]	-	-	-	-	-	-	-	-	-	-	-	-
	CD105	0/2[G⁑,O]	0/1[O]	0/1[O]	0/1[O]	0/1[O]	0/1[O]	0/1[O]	0/1[O]	0/1[O]	0/1[O]	0/1[O]	0/1[O]	0/1[O]
Tumor cell count		0/2[I,J]	0/1[J]	0/1[J]	0/1[J]	0/1[J]	0/1[J]	0/1[J]	0/1[J]	**1/1[J]**	0/1[J]	0/1[J]	0/1[J]	0/1[J]
p53		0/1[D]	0/1[D]	0/1[D†]	0/1[D]	0/1[D]	0/1[D]	0/1[D]	0/1[D]	0/1[D]	0/1[D]	0/1[D]	0/1[D]	0/1[D]
		**V_p**	**DCE-AUC**			**QTM**		**Time-signal intensity curve(TIC)**						
		Mean	60 sec	90 sec	TBF	|υ|	D	RE	ME	MRE	TTP	WIR	WOR	AUC
HPV	All	0/1[C]	0/1[C]	0/1[E]	-	-	-	-	-	-	-	-	-	-
	PCR+P16	-	-	0/1[E]	-	-	-	-	-	-	-	-	-	-
	Other	0/1[C]	0/1[C]	-	-	-	-	-	-	-	-	-	-	-
Ki-67		-	-	-	0/1[F]	**1/1[F]**	0/1[F]	-	-	-	-	-	-	-
HIF-1α		0/1[K]	-	-	**1/1[F]**	**1/1[F]**	0/1[F]	**1/1[L]**	**1/1[L]**	**1/1[L]**	0/1[L]	0/1[L]	0/1[L]	0/1[L]
EGFR		-	-	-	0/1[F]	0/1[F]	0/1[F]	-	-	-	-	-	-	-
VGEF		**1/1[K]**	-	-	-	-	-	-	-	-	-	-	-	
MVD	All	0/1[K]	-	-	-	-	-	-	-	-	-	-	-	0/2[G⁑,i]
	CD31	-	-	-	-	-	-	-	-	-	-	-	-	0/1[I]
	CD34	0/1[K]	-	-	-	-	-	-	-	-	-	-	-	0/1[G]

	CD105	-	-	-	-	-	-	-	-	-	-	-	-	0/1[G]

		**2 Compartment model (2CXM)**					**Arterial spin labeling (ASL)**						
		Fb	PS	Vb	Ve	MTT	Mean	SD	CV	Skewness	Kurtosis	TBF P5	TBF P95
HPV	All	-	-	-	-	-	0/1[A]	**1/1[A]**	0/1[A]	0/1[A]	0/1[A]	0/1[A]	**1/1[A]**
	PCR+P16	-	-	-	-	-	0/1[A]	**1/1[A]**	0/1[A]	0/1[A]	0/1[A]	0/1[A]	**1/1[A]**
	Other	-	-	-	-	-	-	-	-	-	-	-	-
VGEF		**1/1[M]**	0/1[M]	0/1[M]	0/1[M]	0/1[M]	-	-	-	-	-	-	-

		**Contrast Index (CI)**				
		CI max	CI gain max	CI-peak	T-max	CI-gain/CI-max ratio
VGEF		0/1[N]	0/1[N]	0/1[N]	0/1[N]	-
MVD	All	0/2[N,P]	**2/2[N,P]**	0/1[N]	**1/1[N]**	**1/1[P]**
	CD31	0/1[N]	**1/1[N]**	0/1[N]	**1/1[N]**	-
	CD34	0/1[P]	**1/1[P]**	-	-	**1/1[P]**

Table shows the proportion of studies (number significant / number not significant) reporting significant associations between different functional MRI parameters and biological features as indicated. Proportions of more than half testing the indicated association are highlighted in bold.

*Abbreviations = Min: minimum; max: maximum; SD: Standard deviation; P10,P25,P75,P90: percentile 10, 25, 75, 90; Vp: fractional plasma volume; QTM: Quantitative transport mapping; RE: relative enhancement; ME: maximum enhancement; MRE: maximum relative enhancement; TTP: time to peak; WIR: wash-in rate; WOR: wash-out rate; AUC: Area under the curve; Fb: perfusion; PS: Capillary permeability, Vb: blood volume; Ve: Extravascular extracellular space volume; MTT: whole blood mean transit time; CV: Coefficient of variance, TBF: tumor blood flow. Notes: *Significant for P16 positive subgroup analyses; †significant in P16 negative subgroup analyses; ⁂study tested two MVD IHC markers.*

a. Ahn, 2021 (article reference: [25])
b. Choi, 2016 (article reference: [30])
c. Han, 2018 (article reference: [38])
d. Meyer, 2019 (article reference: [48])
e. Piludu, 2021 (article reference: [54])
f. Huang, 2021 (article reference: [40])
g. Karabay, 2022 (article reference: [41])
h. Rasmussen, 2020 (article reference: [60])
i. Surov, 2017 (article reference: [62])
j. Surov, 2018 (article reference: [64])
k. Hu, 2018 (article reference: [39])
l. Lui, 2021 (article reference: [42])
m. Donaldson, 2011 (article reference: [33])
n. Tekiki, 2021 (article reference: [66])
o. Meyer, 2019 (article reference: [50])
p. Unetsubo, 2009 (article reference: [68])
q. Chen Y., 2023 (article reference: [75])

APPENDIX S4 – SIGNIFICANCE OVERVIEW FOR BIOLOGICAL FEATURES WITH LESS THAN FOUR REPORTING SOURCES

Tables depicting the frequency of reported significant results for each individual MRI parameter out of the total number of researched studies, displayed as nr.Significant[significant references]/nr.Total[Other reporting references]. If the percentage of significant studies exceeds 50%, the corresponding numbers are presented in bold font. The tables are sorted by MRI sequence technique: (A4.1) Diffusion parameters (including all IVIM and DKI parameters), (A4.2) Perfusion parameters (including pharmacokinetic models, time-signal intensity curve (TIC), and arterial spin labeling (ASL) parameters), and (A4.3) stand-alone conventional t1w and t2w imaging parameters.

A4.1 STAND-ALONE CONVENTIONAL T1W AND T2W IMAGING PARAMETERS

	**Conventional T1W or T2W (not specified)**		
**Biological factor**	Volume	Diameter	Primary tumor necrosis on MRI
PD-L1	-	-	**1/1[M]**
EBV	0/1[N]	0/1[N]	-

Abbreviations = Min: minimum; max: maximum; SD: Standard deviation; P10,P25,P75,P90: percentile 10, 25, 75, 90

A4.2 DIFFUSION

		**ADC**												
**Biological factor**		Mean	Min	Max	Median	Mode	SD	Kurtosis	Skewness	Entropy	P10	P25	P75	P90
Nuclei Area	Total	**2/2[A,B]**	1[B]/2[A]	0/2[A,B]	**1/1[B]**	0/1[B]	0/1[B]	0/1[B]	0/1[B]	0/1[B]	0/1[B]	**1/1[B]**	0/1[B]	**1/1[B]**
	Average	0/2[A,B]	0/2[A,B]	0/2[A,B]	0/1[B]	0/1[B]	0/1[B]	0/1[B]	0/1[B]	0/1[B]	0/1[B]	0/1[B]	0/1[B]	0/1[B]
HER2		0/2[C,D]	0/1[C]	0/1[C]	0/1[C]	**1/1[C]**	-	0/1[C]	0/1[C]	0/1[C]	0/1[C]	0/1[C]	0/1[C]	0/1[C]
CD3 cell count		0/1[E]	-	-	-	-	-	-	-	-	-	-	-	-
CAIX		0/1[F]	-	-	-	-	-	-	-	-	-	-	-	-
PD-L1		**1/1[F]**	-	-	-	-	-	-	-	-	-	-	-	-
Blc-2		1[F]/2[D]	-	-	-	-	-	-	-	-	-	-	-	-
GLUT-1		0/1[F]	-	-	-	-	-	-	-	-	-	-	-	-
IL6/ IL8/ IL10		0/1[D]	-	-	-	-	-	-	-	-	-	-	-	-
Bak / Bax		0/1[D]	-	-	-	-	-	-	-	-	-	-	-	-

COX-2	0/1[D]	-	-	-	-	-	-	-	-	-	-	-	-
HER2	0/1[C,D]	0/1[C]	0/1[C]	0/1[C]	1/1[C]	0/1[C]	0/1[C]	0/1[C]	0/1[C]	0/1[C]	0/1[C]	0/1[C]	0/1[C]
Tumor stroma ratio	0/1[O]	-	-	-	-	-	-	-	-	-	-	-	-

Abbreviations = Min: minimum; max: maximum; SD: Standard deviation; P10,P25,P75,P90: percentile 10, 25, 75, 90.

A4.3 Perfusion parameters

**Biological factor**		**Volume transfer constant (K^{trans})** Mean	Min	Max	Median	Mode	SD	Kurtosis	Skewness	Entropy	P10	P25	P75	P90
Nuclei Area	Total	0/1[G]	0/1[G]	0/1[G]	0/1[G]	0/1[G]	0/1[G]	0/1[G]	0/1[G]	0/1[G]	0/1[G]	0/1[G]	0/1[G]	0/1[G]
	Average	0/1[G]	**1/1[G]**	0/1[G]	0/1[G]	0/1[G]	0/1[G]	0/1[G]	0/1[G]	0/1[G]	0/1[G]	0/1[G]	0/1[G]	0/1[G]
CAIX		0/2[F,H]	-	-	-	-	-	-	-	-	-	-	-	-
PD-L1		0/1[F]	-	-	-	-	-	-	-	-	-	-	-	-
Blc-2		0/1[F]	-	-	-	-	-	-	-	-	-	-	-	-
GLUT-1		0/1[F]	-	-	-	-	-	-	-	-	-	-	-	-
EBV		0/1[I]	-	-	-	-	-	-	-	-	-	-	-	-
Tumor stroma ratio		0/1[P]	0/1[P]	0/1[P]	0/1[P]	0/1[P]	-	0/1[P]	0/1[P]	**1/1[P]**	0/1[P]	0/1[P]	0/1[P]	0/1[P]
Tumor infiltrating lymphocytes		0/1[P]	0/1[P]	0/1[P]	0/1[P]	0/1[P]	-	0/1[P]	0/1[P]	0/1[P]	0/1[P]	0/1[P]	0/1[P]	0/1[P]
		**Rate constant (K_{ep})**												
		Mean	Min	Max	Median	Mode	SD	Kurtosis	Skewness	Entropy	P10	P25	P75	P90
Nuclei Area	Total	0/1[G]	0/1[G]	0/1[G]	0/1[G]	0/1[G]	0/1[G]	0/1[G]	0/1[G]	0/1[G]	0/1[G]	0/1[G]	0/1[G]	0/1[G]
	Average	0/1[G]	0/1[G]	0/1[G]	0/1[G]	0/1[G]	0/1[G]	0/1[G]	0/1[G]	0/1[G]	0/1[G]	0/1[G]	0/1[G]	0/1[G]
CAIX		0/1[H]	-	-	-	-	-	-	-	-	-	-	-	-
EBV		**1/1[I]**	-	-	-	-	-	-	-	-	-	-	-	-
Tumor stroma ratio		0/1[P]	0/1[P]	0/1[P]	0/1[P]	0/1[P]	-	0/1[P]	0/1[P]	0/1[P]	0/1[P]	0/1[P]	0/1[P]	0/1[P]
		**Extravascular space (V_e)**												
		Mean	Min	Max	Median	Mode	SD	Kurtosis	Skewness	Entropy	P10	P25	P75	P90
Nuclei Area	Total	0/1[G]	0/1[G]	**1/1[G]**	0/1[G]	0/1[G]	0/1[G]	0/1[G]	0/1[G]	**1/1[G]**	0/1[G]	0/1[G]	0/1[G]	0/1[G]
	Average	0/1[G]	0/1[G]	0/1[G]	0/1[G]	0/1[G]	0/1[G]	0/1[G]	0/1[G]	0/1[G]	0/1[G]	0/1[G]	0/1[G]	0/1[G]
CAIX		0/1[H]	-	-	-	-	-	-	-	-	-	-	-	-
EBV		**1/1[I]**	-	-	-	-	-	-	-	-	-	-	-	-
Tumor stroma ratio		0/1[P]	0/1[P]	0/1[P]	0/1[P]	0/1[P]	-	0/1[P]	**1/1[P]**	0/1[P]	0/1[P]	0/1[P]	**1/1[P]**	**1/1[P]**

	**Fractional plasma volume (V_p)**												
	Mean	Min	Max	Median	Mode	SD	Kurtosis	Skewness	Entropy	P10	P25	P75	P90
EBV	0/1[I]	-	-	-	-	-	-	-	-	-	-	-	-
	**Contrast Index (CI)**												
	CI max		CI gain max		CI-peak		T-max	CI-gain/CI-max ratio					
PD-L1	**1/1**[J]		**1/1**[J]		**1/1**[J]		0/1[J]	-					
PCNA	1[L]/2[K]		**2/2**[K,L]		-		-	**1/1**[K]					

Abbreviations = Min: minimum; max: maximum; SD: Standard deviation; P10,P25,P75,P90: percentile 10, 25, 75, 90.

Notes: †significant in P16 negative subgroup analyses. Abbreviations = SD: standard deviation; P10/25/75/90: percentile

a. Surov, 2016 (article reference: [61])
b. Surov, 2018 (article reference: [63])
c. Meyer, 2018 (article reference: [47])
d. Tse, 2010 (article reference: [67])
e. Swartz, 2018 (article reference: [65])
f. Rasmussen, 2020 (article reference: [60])
g. Surov, 2018 (article reference: [64])
h. Newbold, 2009 (article reference: [80])
i. Sriyook, 2021 (article reference: [78])
j. Tekiki, 2021 (article reference: [66])
k. Unetsubo, 2009 (article reference: [68])
l. Konouchi, 2003 (article reference: [79])
m. Chen T., 2015 (article reference: [31])
n. Samolyk-Kogaczewska, 2020 (article reference: [56])
o. Choi, 2017 (article reference: [81])
p. Meyer, 2021 (article reference: [82)]
